# Genetic Dissection of Triple Rust Resistance (Leaf, Yellow, and Stem Rust) in Kenyan Wheat Cultivar, “Kasuku”

**DOI:** 10.3390/plants14071007

**Published:** 2025-03-23

**Authors:** Naeela Qureshi, Ravi Prakash Singh, Sridhar Bhavani

**Affiliations:** 1 International Maize and Wheat Improvement Center (CIMMYT), Carretera Mexico-Veracruz Km. 45, El-Batan, Texcoco 56237, Mexico; r.singh@cgiar.org; 2 International Maize and Wheat Improvement Center (CIMMYT), ICRAF Campus, United Nations Avenue, Gigiri, Nairobi P.O. Box 1041-00621, Kenya; s.bhavani@cgiar.org

**Keywords:** wheat rusts, quantitative trait loci (QTL), genetic mapping, wheat breeding, sustainable agriculture

## Abstract

Climate change is driving the spread of transboundary wheat diseases, necessitating the development of resilient wheat varieties for sustainable agriculture. Wheat rusts, including leaf rust (LR), yellow rust (YR), and stem rust (SR), remain among the most economically significant diseases, causing substantial yield losses worldwide. Enhancing genetic diversity by identifying and deploying rust resistance genes is crucial for durable resistance in wheat breeding programs. This study aimed to identify quantitative trait loci (QTL) associated with rust resistance in the CIMMYT wheat line Kasuku, released in Kenya in 2018. A recombinant inbred line (RIL) population (181 lines) derived from Kasuku (triple rust-resistant) and Apav#1 (triple rust-susceptible) was evaluated under artificial LR and YR epidemics in Mexico and YR and SR in Kenya. QTL mapping using genotyping-by-sequencing (DArTSeq) and phenotypic data identified four major loci: *QLrYrSr.cim-1BL* (*Lr46*/*Yr29*/*Sr58*) on 1BL, conferring resistance to LR, YR, and SR; *QLrYr.cim-2AS* (*Yr17*/*Lr37*) on 2AS, providing LR and YR resistance; *QLrYr.cim-3AL* on 3AL; and *QLrYrSr.cim-6AL* on 6AL, representing novel loci associated with multiple rust resistances. Additionally, minor QTL were also identified: for LR (*QLr.cim-2DS* on 2DS, *QLr.cim-6DS* on 6DS), for YR (*QYrKen.cim-3DS* on 3DS, *QYrKen.cim-6BS* on 6BS), and for SR (*QSr.cim-2BS* on 2BS, *QSr.cim-5AL* on 5AL, *QSr.cim-6AS* on 6AS). RILs carrying these QTL combinations exhibited significant reductions in rust severity. Flanking markers for these loci are being used to develop Kompetitive Allele-Specific PCR (KASP) markers for fine mapping and marker-assisted selection (MAS). These findings contribute to the strategic deployment of rust resistance genes in wheat breeding programs, facilitating durable resistance to multiple rust pathogens.

## 1. Introduction

Rust pathogens of wheat are among the most destructive pathogens known to cause serious threats to crop production [1]. The three rusts species, yellow rust, caused by *Puccinia striiformis* f. sp. *tritici* (*Pst*), leaf rust, caused by *Puccinia triticina* (*Pt*), and stem rust, caused by *Puccinia graminis* f. sp. *tritici* (*Pgt*), are known to cause global losses equivalent to post-World War II in wheat [2]. To mitigate the effect of rust diseases, chemical control and genetic control have always been widely used [3]. Both strategies face the risk of rust pathogens developing resistance to fungicides, and this resistance could potentially be exacerbated by the loss of avirulence in the resistance gene [4]. Deploying genetic resistance is favored for rust control due to its environmental and economic benefits [5]. Genetic control can either be achieved by deploying race-specific all-stage resistance (ASR), usually controlled by one or few genes with major effects, and/or non-race specific resistance, also referred to as quantitative disease resistance or adult plant resistance (APR), controlled by multiple genes with relatively smaller effects [6,7,8,9]. The deployment of a single effective ASR gene in any cultivar is often followed by the breakdown or loss of effectiveness of resistance due to gain of virulence in the pathogen [10]. APR is proven to provide more durable resistance because of the additive effects of several different genes, each exerting small selection pressure on the pathogen [11]. Therefore, strategic deployment combining both ASR and APR genes in wheat cultivars, along with a comprehensive understanding of current pathogenic variations and identification of genetically diverse sources of resistance, are recommended strategies to achieve sustainable rust control [6,12,13].

Pleiotropic or co-located loci refers to a phenomenon where a single genetic locus can have distinct linked genes, each associated with resistance to different diseases, or an individual allele that confers resistance to multiple diseases. Pleiotropic resistance genes to various diseases in wheat has been documented, such as *Lr34*/*Yr18*/*Sr57*/*Pm38*/*Bdv1*/*Sb1*/*Ltn1*, which provides broad-spectrum resistance to leaf rust (LR), yellow rust (YR), stem rust (SR), powdery milder (PM), barley yellow dwarf virus (BYDV), and spot blotch diseases [14,15,16,17,18,19]. Similarly, *Lr46*/*Yr29*/*Sr58*/*Pm39*/*Ltn2* [20] and *Lr67*/*Yr46*//*Sr55*/*Pm46*/*Ltn3* [21] also show resistance to rust and mildew diseases. All these genes are also linked to the leaf tip necrosis (LTN) phenotype, which serves as a morphological marker on flag leaves when grown in the field [18,22,23,24,25]. In addition to these pleiotropic loci, *Sr2*, an APR gene for SR transferred from tetraploid Yaroslav emmer to the susceptible bread wheat variety “Marquis” in the 1920s [26] is also likely pleiotropic, conferring partial YR resistance, *Yr30*, and PM resistance, *Pm48*. *Sr2* is also linked to the pseudo-black chaff (PBC) phenotype and serves as a morphological marker in the field. All these APR genes provide partial rust resistance, and their expression is highly influenced by the environment. Partial rust resistance is not adequate to avoid yield losses, especially under high disease pressure, unless these genes are present in combination with additional ASR and/or APR genes [27,28].

The interest in quantitative resistance conferring durable resistance to one and/or all three rust diseases around the world has accelerated the discovery and characterization of new quantitative trait loci (QTL) to overcome the ever-evolving nature of rust pathogens. QTL mapping is one of the widely used methods for the genetic dissection of complex quantitative traits in crops. It involves studying bi-parental populations under diverse environmental conditions across different seasons to identify genomic regions associated with these traits.

The advancements in next-generation sequencing technologies have significantly enhanced the ability to dissect rust resistance genetics with greater precision. The advancements in next-generation sequencing (NGS) technologies have significantly enhanced the ability to dissect rust resistance genetics with greater precision. Various high-throughput genotyping platforms, such as whole-genome sequencing (WGS), genotyping-by-sequencing (GBS) [29], high-density SNP arrays, e.g., Illumina 9K [30] and 90K [31] SNP arrays, and Diversity Arrays Technology sequencing (DArTSeq) [32], have been widely used in genetic studies. While WGS provides the most comprehensive genomic information, it is often cost-prohibitive for large populations.

DArTSeq, a form of reduced-representation sequencing, provides cost-effective, high-density genome-wide marker coverage, making it an efficient tool for mapping complex traits such as rust resistance in wheat. CIMMYT benefits from its in-house SAGA platform in the Biotech lab, which offers subsidized DArTSeq genotyping, enabling large-scale genetic analysis at reduced costs while maintaining data accuracy. These advantages make DArTSeq a powerful tool for advancing rust resistance breeding strategies.

Therefore, this study aims to identify and genetically characterize resistance loci within Kasuku, a high-yielding wheat variety from Kenya (KENYA SWARA/SAUAL//SAUAL/3/BORLAUG100 F2014) developed and released by CIMMYT in 2016 and by the Kenyan Agricultural and Livestock Research Organization (KALRO) in 2018. Kasuku stands out for its triple rust resistance traits, tailored to thrive in diverse environments found in both Mexico and Kenya.

## 2. Results

### 2.1. Evaluation of Stripe Rust and Leaf Rust Resistance at the Seedling Stage

**Figure 1:**
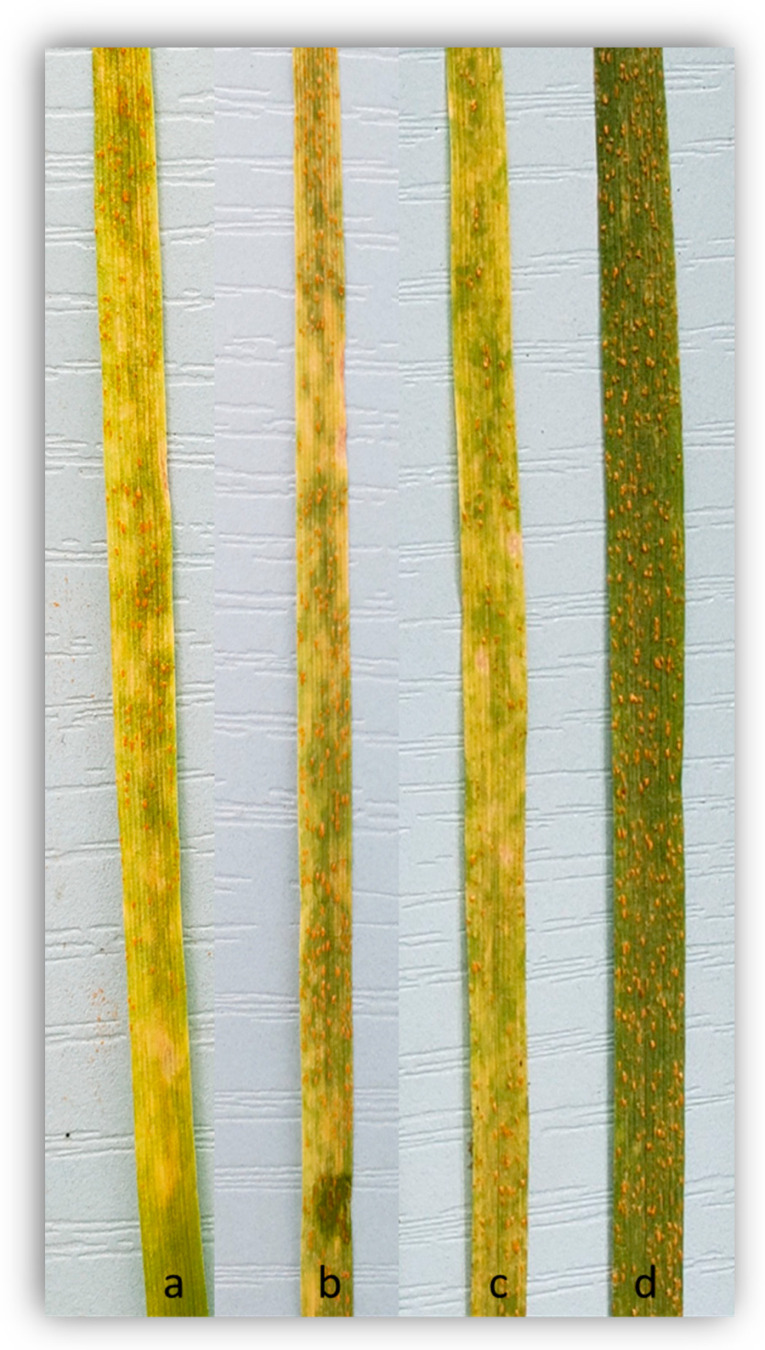
Seedling infection type responses to *Puccinia striiformis* race MEX14.191: (**a**) resistant parent (Kasuku, IT 23C), (**b**,**c**) homozygous resistant RILs (IT 23C), and (**d**) susceptible parent (Apav#1, IT 4).

Seedlings of the resistant parental line Kasuku showed an infection type (IT) of 23C to *Pst* MEX14.191, while Apav#1 had a high IT of 4 to MEX14.191 *Pst* race on a 0–4 disease rating scale (Figure 1). The homozygous resistant (HR) RILs produced IT of 23C to 3C, while the homozygous susceptible RILs exhibited an IT of 4 (Figure 1). The seedling response of 181 RILs showed segregation for a single resistance locus (80 resistant:101 susceptible, *χ*^2^_1:1_ = 2.44, *P_1d.f_* > 0.05). The locus was temporarily named *YrKasuku*.

The entire RIL population of Kasuku/Apav#1 was susceptible when tested at the seedling stage against the *Pt* race, MBJ/SP.

### 2.2. Evaluation of Stripe Rust, Leaf Rust, and Stem Resistance at the Adult Plant Stage

**Figure 2:**
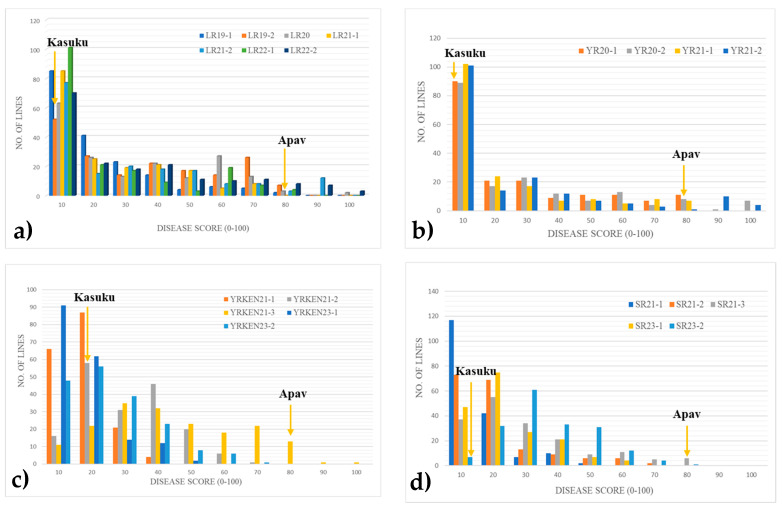
Frequency distribution of the Kasuku/Apav#1 RIL population analyzed across various environments under natural conditions: (**a**) leaf rust in El-Batan and Obregon, Mexico; (**b**) stripe rust in Toluca, Mexico; (**c**) stripe rust in Njoro, Kenya; and (**d**) stem rust in Njoro, Kenya.

The evaluation of all three rusts at the adult plant stage revealed high disease pressure across all locations and years. For LR, the resistant parent Kasuku demonstrated an average disease severity of 5, whereas the susceptible parent Apav#1 displayed an average disease severity of 80 over four years (Figure 2a). In Mexico, Kasuku exhibited an average stripe rust disease severity of 0 over two years, while Apav#1 had an average disease severity of 86.2 (Figure 2b). These severity scores were based on the 0–100 modified Cobb disease rating scale. The severity scores for YR and LR were significantly correlated across the years, with correlation coefficients ranging from r = 0.85 to 0.96 for stripe rust and r = 0.71 to 0.90 for LR, indicating minimal environmental variation in overall disease pressure between environments such as El-Batan and Obregon (Table 1).

In Kenya, Kasuku and Apav#1 exhibited average stripe rust disease severities of 15 and 80, respectively (Figure 2c). For stem rust, Kasuku showed an average disease severity of 5, while Apav#1 had an average severity of 80 (Figure 2d). The correlation coefficients for YR and SR disease severities ranged from r = 0.75 to 0.94 and r = 0.84 to 0.94, respectively, indicating significant correlation across years (Table 2).

### 2.3. Environmental Influence on Disease Development

While differences in disease severity across environments were minimal, the role of environmental factors is critical in the development and spread of rust diseases. Favorable environmental conditions, such as moderate temperatures, high humidity, and prolonged leaf wetness, drive the rapid onset and progression of rust epidemics. Consistently high disease pressure across locations and years suggests that conditions in both Obregon and El-Batan were conducive to disease development, minimizing the apparent environmental variation in disease severity scores. These locations are part of CIMMYT’s phenotyping platforms, which are strategically chosen as rust disease hotspots to provide optimal conditions for consistent and reliable disease evaluation.

The frequency distribution of mean disease severities of RILs showed continuous variation in all environments against YR, LR, and SR, suggesting a mostly quantitative basis of rust resistance. The strong correlation in LR severities between Obregon and El-Batan indicates that stable resistance QTLs are consistently expressed across environments when favorable conditions for disease development are present. This highlights the importance of quantitative resistance in managing diseases under diverse yet conducive environments.

While environmental factors influence disease onset and spread, the high correlation in disease severities across locations underscores the stability of QTL expression in Kasuku. These findings suggest that favorable conditions for disease development may override subtle environmental differences, leading to consistent disease pressure and robust performance of resistant genotypes. The use of CIMMYT’s phenotyping platforms as hotspots further ensures that disease resistance is evaluated under highly challenging conditions, thereby increasing the reliability of resistant lines such as Kasuku in breeding programs aimed at developing durable rust resistance.

### 2.4. Linkage Map Construction

The Kasuku/Apav#1 RIL population was genotyped using the DArTSeq platform. A linkage map consisting of 8281 non-redundant markers showing Mendelian segregation was constructed using 181 RILs. Forty-three linkage groups were generated, representing all 21 wheat chromosomes, with the B genome having the highest number of markers and the D genome having the lowest number of markers due to its lower genetic diversity, restricted gene pool, and lower recombination frequency compared to the A and B genomes, which have undergone extensive breeding and selection.

### 2.5. Significant QTL Across Environments

#### 2.5.1. Co-Located Rust Resistance QTL

Significant QTL were identified based on a LOD threshold of 3 or higher and their consistency across different environments. Composite interval mapping (CIM) using QTL Cartographer identified two highly significant and consistent co-located QTL across multiple years for triple rust resistance. These QTL were detected on chromosome 1BL and 6AL: *QLrYrSr.cim-1BL* and *QLrYrSr.cim-6AL*, respectively. Additionally, another two significant and stable co-located QTL for LR and YR resistance were identified on chromosomes 2AS and 3AL: *QLrYr.cim-2AS* and *QLrYr.cim-3AL*, respectively.

**Figure 3:**
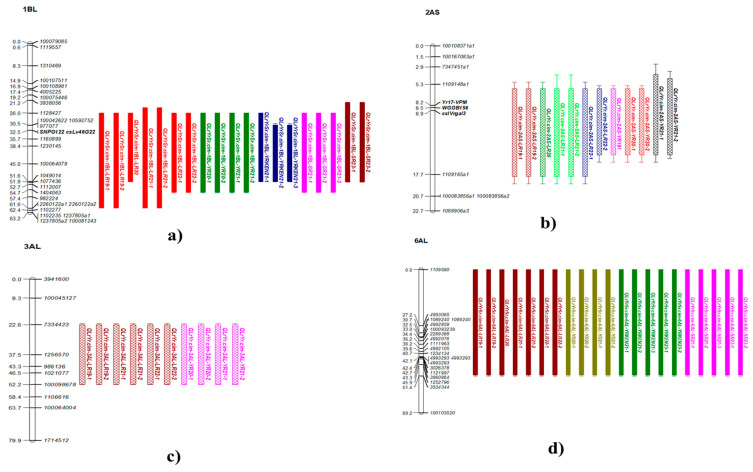
Linkage maps showing QTL regions identified using inclusive composite interval mapping for triple rust resistance response: (**a**) *QLrYrSr.cim-1BL* (chromosome 1BL), (**b**) *QLrYr.cim-2AS* (chromosome 2AS), (**c**) *QLrYrSr.cim-6AL* (chromosome 6AL), and (**d***) QLrYr.cim-3AL* (chromosome 3AL). The figures display only chromosome segments with QTL and flanking markers, with genetic distances (cM) on the left. LOD values and physical positions are detailed in Supplementary Material—Table S1. *QLrYr.cim-2AS* was another most stable pleiotropic QTL identified in all environments in Mexico with 5 to 59% of PVE and LOD varying from 5.6 to 47.7% across various environments (Table 3; Supplementary Material—Table S1). It was not detected with the datasets from the Kenyan environment for both YR and SR. *QLrYr.cim-2AS* peaked between DArTSeq markers *3024467* and *4993397* (**b**) and was positioned around 6–9 Mb of chromosome 2AS on the IWGSC_RefSeq_v1.0 assembly [33]. The resistant parent, Kasuku, was also the source of resistant alleles of this QTL. The seedling disease score was also incorporated into the QTL mapping, and it peaked at the same genomic location as observed with other YR datasets, thereby confirming that *YrKasuku* corresponded to *QLrYr.cim-2AS*. VPM1 resistance, known to carry *Yr17*, *Lr37,* and *Sr38* resistance genes, is also located on the 2NS/2AS translocation, which corresponds to the approximately 32.6–33.5 Mb region on the short arm of the 2A chromosome. *Yr17*/*Lr37*/*Sr38* linked markers, *WGGB156*, *cslVrgal3*, and VPM SNP, were also genotyped on the bi-parental population to confirm if *QLrYr.cim-2AS* corresponded to the same locus. All these markers were added to the linkage map and were mapped between the linked DArTSeq markers to *QLrYr.cim-2AS*.

The pleiotropic QTL, *QLrYrSr.cim-1BL,* was contributed by the resistant parent, Kasuku, with 4–22% PVE and logarithms of the odds (LOD) scores ranging from 4.4 to 26.9 across different environments (Table 3; Supplementary Material—Table S1). QTL *QLrYrSr.cim-1BL* was delimited between DArTSeq markers *100107511* and *2260122* (Figure 3a) and was mapped around 678–685 Mb on the IWGSC_RefSeq_v1.0 assembly of Chinese Spring [33]. According to the IWGSC [33] physical assembly information of CS, there is a pleiotropic locus located around the same position of about 670–680 Mb, known as *Lr46*/*Yr29*/*Sr58*/*Pm39*. To confirm if *QLrYrSr.cim-1BL* corresponds to the *Lr46*/*Yr29*/*Sr58*/*Pm39* locus, the bi-parental population was genotyped and validated using the closely linked markers, which were then added to the genetic map.

*QLrYr.cim-3AL* represented another co-located locus on chromosome 3AL, conferring resistance against both LR and YR, with 5–8.8% of the PVE. This locus demonstrated stability across multiple years and diverse environments in Mexico. It spanned the genetic interval between DArTSeq markers *7334423* and *100098678*, covering the physical region around 747 Mb on the IWGSC_RefSeq_v1.0 assembly (Supplementary Material—Table S1; Figure 3c).

Another co-located QTL for triple rust resistance, *QLrYrSr.cim-6AL,* was identified on chromosome 6AL. This QTL exhibited consistency across different environments and years in both Mexico and Kenya. In Mexico, 7.3–11.7% and 7.5–9.5% of PVE for LR and YR resistance, respectively, was observed. In Kenya, it contributed 4–6% of PVE for YR resistance and accounted for 5–7.7% of PVE for SR resistance across all datasets. It spanned approximately 609–614 Mb on the IWGSC_RefSeq_v1.0 assembly [33] and was mapped between *1109580* and *3534344* DArTSeq markers (Table 3; Figure 3d; Supplementary Material—Table S1).

#### 2.5.2. Other Minor QTL for Leaf Rust, Yellow Rust, and Stem Rust

**Figure 4:**
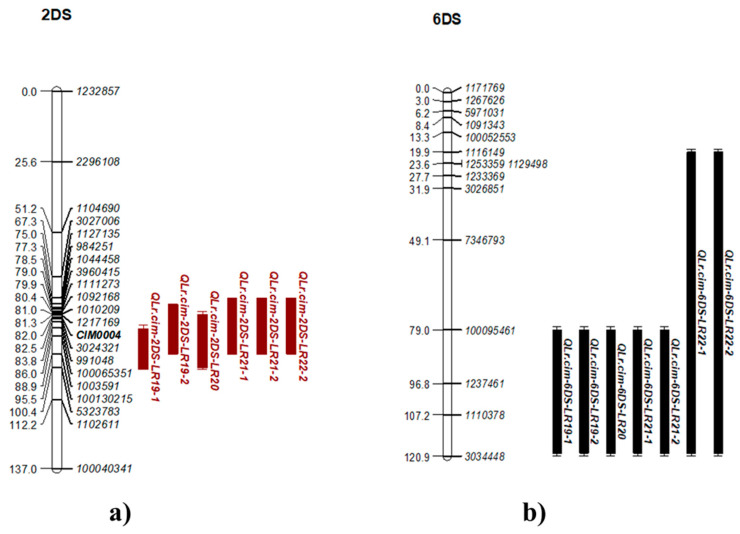
Linkage maps showing QTL regions for minor leaf rust resistance: (**a**) *QLr.cim-2DS* (2DS) and (**b**) *QLr.cim-6DS* (6DS). The figures display chromosome segments with QTL and flanking markers based on genetic distances (cM). LOD values and physical positions are in Supplementary Material—Table S1.

Several other additional minor QTL were identified in the population. In addition to *QLrYrSr.cim-1BL*, two other consistent minor QTL for LR, *QLr.cim-2DS* and *QLr.cim-6DS,* were also identified on chromosome 2DS and 6DS, respectively. *QLr.cim-2DS* and *QLr.cim-6DS* were the most stable QTL, with almost all the LR datasets having <10% of the PVE (Table 3). *QLr.cim-2DS* mapped between markers *1104690* and *1102611* (Table 3; Figure 4a; Supplementary Material—Table S1) was between 12–17 Mb on the IWGSC_RefSeq_v1.0 assembly [33], whereas *QLr.cim-6DS* was detected on the chromosome 6DS within the marker interval of *1116149* and *3034448* spanning 7–19 Mb on the IWGSC_RefSeq_v1.0 assembly (Figure 4b; IWGSC 2018).

**Figure 5:**
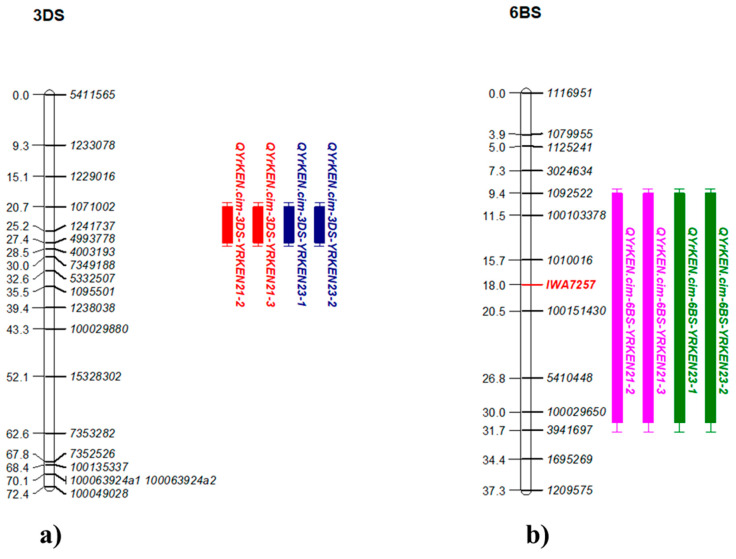
Linkage maps for minor QTL associated with stripe rust resistance in Kenya: (**a**) *QYr.cim-3DS* on chromosome 3DS and (**b**) *QYr.cim-6BS* on chromosome 6BS. The figures display chromosome segments with QTL and flanking markers based on genetic distances (cM). LOD values and physical positions are in Supplementary Material—Table S1.

Two QTL were identified for YR resistance in Kenya on chromosomes 3DS and 6BS, *QYrKen.cim-3DS* and *QYrKen.cim-6BS,* with minor effects of less than 10% PVE (Supplementary Material—Table S1). QTL *QYrKen.cim-3DS* was identified between the marker interval of *1071002*–*4993778* (Figure 5a; Supplementary Material—Table S1), and *QYrKen.cim-6BS* was identified between the markers *1109174* and *1137936* (Figure 5b; Supplementary Material—Table S1). Marker *CIM0004,* linked to another QTL on chromosome 2DS [34] around the same position as the QTL in the current study, was also genotyped on the population and was added to the genetic map.

**Figure 6:**
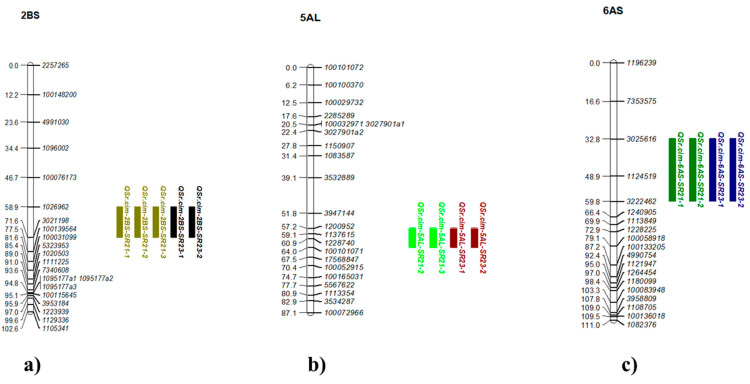
Linkage maps for minor QTL associated with stem rust resistance in Kenya: (**a**) *QSr.cim-2BS* on chromosome 2BS, (**b**) *QSr.cim-5AL* on chromosome 5AL, and (**c**) *QSr.cim-6AS* on chromosome 6AS. The figures display chromosome segments with QTL and flanking markers based on genetic distances (cM). LOD values and physical positions are in Supplementary Material—Table S1.

Three more minor QTL were detected for the stem rust resistance on chromosomes 2BS (*QSr.cim-2BS*), 5AL (*QSr.cim-5AL*), and 6AS (*QSr.cim-6AS*). *QSr.cim-2BS* and *QSr.cim-5AL* had less than 10% PVE, while *QSr.cim-6AS* had 9–12% of PVE (Table 3; Figure 6a–c; Supplementary Material—Table S1). All the QTL were contributed by Kasuku.

### 2.6. Cumulative Interaction of Multiple QTL

Flanking markers adjacent to the QTL regions were used to identify RILs harboring various combinations of QTL. It was observed that the additive effects of multiple QTL resulted in RILs expressing very low levels of mean disease severity (MDS; 5%), while those carrying few to none of any QTL showed higher (50–80%) MDS on a 0–100 disease rating scale.

**Figure 7:**
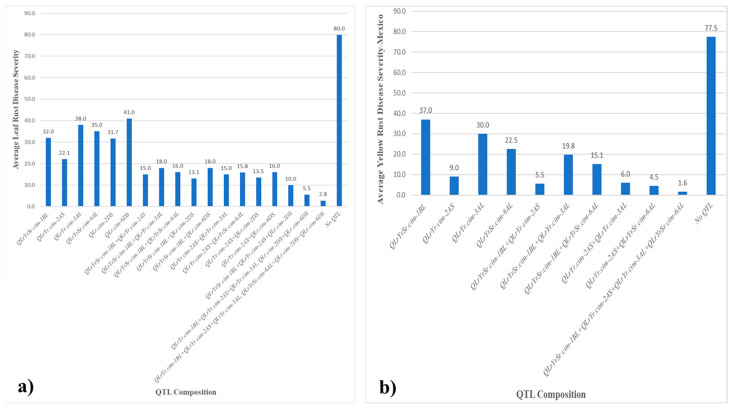
Mean disease severity (MDS) among RILs with various QTL combinations: (**a**) leaf rust in Mexico and (**b**) stripe rust in Mexico.

For LR resistance, RILs carrying the combination of QTL (*QLrYr.cim-1BL*, *QLrYr.cim-2AS*, *QLrYr.cim-3AL*, *QLrYrSr.cim-6AL*, *QLr.cim-2DS*, and *QLr.cim-6DS*) demonstrated a significantly lower MDS of 2.8. In contrast, when these QTL were present individually, the MDS values were higher: *QLrYr.cim-1BL* (32 MDS), *QLrYr.cim-2AS* (22.1 MDS), *QLrYr.cim-3AL* (38 MDS), *QLrYrSr.cim-6AL* (35 MDS), *QLr.cim-2DS* (31.7 MDS), and *QLr.cim-6DS* (41 MDS). RILs carrying QTL in dual combinations also exhibited much lower MDS values, whereas those without any QTL showed a considerably higher MDS of 80 (Figure 7a).

For YR in Mexico, when QTL were present individually, RILs having those QTL exhibited varying degrees of effectiveness in reducing disease severity: *QLrYrSr.cim-1BL* had an MDS of 37.0, *QLrYr.cim-2AS* showed an MDS of 9.0, *QLrYr.cim-3AL* was at 30.0 MDS, and *QLrYrSr.cim-6AL* scored 22.5. These MDS indicated moderate to low levels of disease severity when these QTL were individually active. On the other hand, combinations of QTL resulted in significantly lower disease scores, highlighting their additive or synergistic effects. For instance, *QLrYrSr.cim-1BL* + *QLrYr.cim-2AS* reduced the MDS to 5.5, *QLrYrSr.cim-1BL* + *QLrYr.cim-3AL* to 19.8, *QLrYrSr.cim-1BL* + *QLrYrSr.cim-6AL* to 15.1, *QLrYr.cim-2AS* + *QLrYr.cim-3AL* to 6.0, and *QLrYr.cim-2AS* + *QLrYrSr.cim-6AL* to 4.5. The combination of *QLrYrSr.cim-1BL* + *QLrYr.cim-2AS* + *QLrYr.cim-3AL* + *QLrYrSr.cim-6AL* exhibited the lowest score of 1.6, indicating a high level of resistance. In contrast, RILs lacking any QTL demonstrate a significantly higher disease score of 77.5 (Figure 7b).

**Figure 8:**
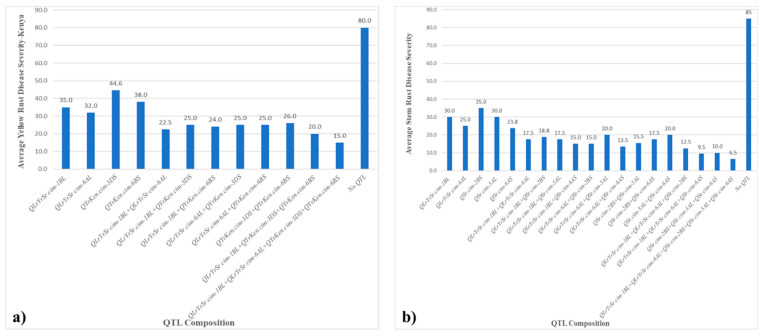
Mean disease severity (MDS) among RILs with various QTL combinations: (**a**) stripe rust in Kenya and (**b**) stem rust in Kenya.

For YR in Kenya, RILs carrying various QTL compositions exhibited different levels of effectiveness in reducing disease severity: *QLrYrSr.cim-1BL* scored an MDS of 35, *QLrYrSr.cim-6AL* scored 32, *QYrKen.cim-3DS* scored 44.6, and *QYrKen.cim-6BS* scored 38.0. These MDS scores indicated moderate levels of disease severity when these QTL were present individually. On the contrary, combinations of QTL generally resulted in lower disease scores, highlighting their combined impact. For instance, *QLrYrSr.cim-1BL* + *QLrYrSr.cim-6AL* reduced the MDS to 22.5, *QLrYrSr.cim-1BL* + *QYrKen.cim-3DS* to 25.0, *QLrYrSr.cim-1BL* + *QYrKen.cim-6BS* to 24.0, *QLrYrSr.cim-6AL* + *QYrKen.cim-3DS* to 25.0, *QLrYrSr.cim-6AL* + *QYrKen.cim-6BS* to 25.0, *QYrKen.cim-3DS* + *QYrKen.cim-6BS* to 26.0, *QLrYrSr.cim-1BL* + *QYrKen.cim-3DS* + *QYrKen.cim-6BS* to 20, and *QLrYrSr.cim-1BL* + *QLrYrSr.cim-6AL* + *QYrKen.cim-3DS* + *QYrKen.cim-6BS* to 15.0. RILs lacking any QTL demonstrated a significantly higher disease score of 80.0 (Figure 8a).

For SR, RILs that carried QTL singly showed various MDS: *QLrYrSr.cim-1BL* exhibited an MDS of 30.0, *QLrYrSr.cim-6AL* of 25.0, *QSr.cim-2BS* of 35.0, *QSr.cim-5AL* of 30.0, and *QSr.cim-6AS* of 23.8. In contrast, when QTL were present in combination, there was a notable reduction in disease severity. Combinations such as *QLrYrSr.cim-1BL* + *QLrYrSr.cim-6AL* resulted in an average disease score of 17.5, *QLrYrSr.cim-1BL* + *QSr.cim-2BS* at 18.8, *QLrYrSr.cim-1BL* + *QSr.cim-5AL* at 17.5, and *QLrYrSr.cim-1BL* + *QSr.cim-6AS* at 15.0, demonstrating additive effects in reducing disease severity. Similarly, *QLrYrSr.cim-6AL* combined with *QSr.cim-6AS* showed an MDS of 13.5. The lowest disease severity was observed in combinations involving multiple QTL, such as *QLrYrSr.cim-1BL* + *QLrYrSr.cim-6AL* + *QSr.cim-2BS* + *QSr.cim-5AL* + *QSr.cim-6AS,* with an MDS of 6.5. In contrast, lines lacking any QTL exhibited the highest disease severity with an average score of 85 (Figure 8b).

This illustrated how different additive combinations of QTL contribute to varying levels of disease resistance, with combined QTL generally resulting in lower disease severity compared to individual QTL or no QTL present.

## 3. Discussion

The continuous evolution of rust pathogens around the globe has limited the availability of genes that confirm broad effectiveness across multiple environments, hindering the development of wheat cultivars with durable resistance. This underscores the importance of identifying and characterizing new sources of durable resistance against rust pathogens to ensure their effective use in wheat-breeding programs. Rust resistance plays a crucial role in the CIMMYT breeding program for developing advanced germplasm with a high level of resistance that provides improved wheat nurseries combining yield, broad adaptation, and biotic and abiotic stress tolerance to partners in multiple countries. In this study, we identified four pleiotropic/co-located loci and multiple other genomic regions associated with APR in both Mexican and Kenyan environments in a Kenyan variety “Kasuku”, developed by the collaboration of KALRO and CIMMYT.

### 3.1. Co-Located QTL Regions

#### 3.1.1. Genomic Regions Associated with Triple Rust Resistance

The QTL on 1BL linked with triple rust resistance, *QLrYrSr.cim-1BL*, exhibited one of the highest percentages of PVE in the bi-parental RIL population and was mapped to the distal end of the chromosome, around 678–685 Mb on the IWGSC_RefSeq_v1.0 assembly [33]. This QTL consistently demonstrated stability across different years and locations, highlighting its significance. In CIMMYT, the wheat germplasm *Lr46*, located on the long arm of chromosome 1B, is a prevalent slow rusting APR gene and has been a significant contributor to durable rust resistance for more than 60 years [35,36,37]. This locus also imparts broad-spectrum resistance against YR (*Yr29*) and SR (*Sr58*) [20]. Furthermore, it is associated with the morphological marker LTN [38]. Based on the wide distribution of this gene, the position of flanking markers, and the expression of LTN in our RIL population, *QLrYrSr.cim-1BL* was confirmed to be *Lr46*/*Yr29*/*Sr58*. To further substantiate this identification, the entire RIL population was genotyped using the KASP marker *SNPG122* and *csLv46G22*, known to be closely linked to the gene *Lr46*/*Yr29*/*Sr58*, which confirmed the results. The interaction of *Lr46*/*Yr29*/*Sr58* with other QTL was significant in reducing disease severity, which aligns with previous studies that, when present in combination, *Lr46*/*Yr29*/*Sr58* has an additive effect as compared to when present on its own [34].

Another co-located resistance QTL showing triple rust resistance, *QLrYrSr.cim-6AL,* was flanked by the DArTSeq markers *1109580* and *3534344*, corresponding to the 609–614 Mb on the IWGSC_RefSeq_v1.0 assembly. Various resistance genes and QTL were previously reported on chromosome 6AL, such as *Yr38* linked to *Lr56* from *Aegilops sharonensis* [39], *Yr42* linked to *Lr62* from *Aegilops neglecta* [40], *YrHua* from *Psathyrostachys huashanica* accession 0503383 [41], and *Lr64* from *Triticum dicoccoides* [42], mapped to the telomeric region of the chromosome, differ in their mode of resistance and source of origin, suggesting *QLrYrSr.cim-6AL* to be different. *QLr.cim-6AL*/*QYr.cim-6AL* [43] was reported as a co-located QTL for both LR and YR in the vicinity of DArT-array markers *wPt-4229*, *wPt-6829*, and *wPt-8954,* which are located around 611.4 Mb of the Triticum_aestivum_IWGSC_RefSeq_v1.0_DArT_ver3_markers (https://plantinformatics.io/: accessed on 21 August 2024), suggesting it to be a similar QTL for LR and YR. Quite a few other studies also reported adjacent or overlapping QTL within the same interval for YR. Ref. [44] reported a QTL linked to markers, *Tdurum_contig29607_413* at 609.4 Mbp and *GENE-4021_496* at 610 Mb. *QYr.nmbu.6A* was mapped within the marker interval of 610–612 Mb [45] with the same significant marker, *GENE-4021_496,* as reported by [44], and [46] also detected a locus on 6AL associated with adult plant YR resistance linked to *Tdurum_contig29607_413* as a significant marker. Furthermore, Ref. [47] identified a QTL on 6AL of the Attraktion genome within the marker interval of 611.98 Mb, which corresponds to around 610 Mb on the CS IWGSC RefSeq v1.0. Several other QTL have been mapped on chromosome 6A, including *QYr.niab-6A.3* at 596.5 Mb [48], *Qyr.gaas.6A* spanning 609.11–609.89 Mb [49], and *YrLM168*, which is lo-cated near the same interval as Qyr.gaas.6A [50,51]. Additional QTL such as *QYr-6A_Saar* [17], *QYrpl.orr-6AL_Stephens* [52], *QYr-6A_Avocet* [53], and *QYrtb.orz-6AL* at 611.4 Mb [54] were mapped with linkage to the same marker (*wPt-4229*) as *QLr.cim-6AL/QYr.cim-6AL*, suggesting that all these QTL may represent the same genetic locus. Most of these QTL have been identified in European winter wheat germplasm, except for *QYr.nmbu.6A,* which was reported in spring wheat material.

Not many LR QTL have been previously reported on chromosome 6AL [28]. Two QTL associated with LR, *QLr.cim-6AL* [53] and *QLr.Hbau-6AL* [55], were mapped on the middle of the chromosome 6AL based on linked markers, whereas *QLr.cim-6AL*, also linked to YR resistance QTL, *QYr.cim-6AL,* was reported by [43] located around 611 Mb on the IWGSC_RefSeq_v1.0 assembly and is most likely to be the same QTL in our study. Another QTL, *QLr.ramp-6A.4,* was also mapped within the marker interval of 614.17–615.46 and was considered to be strongly associated with *Lr64* [56].

Two major ASR genes, *Sr13* (*Sr13a*, *Sr13b*, *Sr13c*) and *Sr67,* are reported to be located approximately within the marker interval of around 615.45 Mb [57] and 616.4 Mb [58], respectively. Seedling tests on the parental lines revealed susceptibility to all Ug99 races except TTKSK. The PVE in our study was minimal, indicating that this QTL does not classify as a major QTL. Additionally, *QSr.ramp-6A.5* was identified through the GWAS study and was mapped to the marker interval approximately spanning 605–615 Mb on chromosome 6AL [56], possibly overlapping with the *Sr13* locus. QTL *QSr_NCCR.6A.2* was mapped between 591.6–602.7 Mb and may correspond to the loci reported at 598.6 Mb by [59]. The QTL *QSr_CxN.6A.2* was detected at the same marker interval of *Sr13*, whereas *QSr_NCCR.6A.3* was detected 2 Mb distal to the *Sr13* locus and was suggested to may be representing another allele of *Sr13* [60]. All these reported QTL are associated with the ASR category, whereas the QTL detected in the current study was consistent across environments but with minimal PVE.

#### 3.1.2. Genomic Regions Associated with Dual Rust Resistance

Co-located loci, *QLrYr.cim-2AS*, showing dual rust resistance to LR and YR, was mapped between 5–16 cM, corresponding to approximately 6–9 Mb on the IWGSC_RefSeq_v1.0 assembly, with 15–59% of the PVE. This high PVE is attributed to *QLrYr.cim-2AS* being a major gene, showing 2C to 23C IT under controlled conditions, also consistent with seedling resistance *YrKasuku*. *Yr17*/*Lr37*/*Sr38* is a resistance gene located on wheat chromosome 2AS carrying the translocation of the *Aegilops ventricosa* 2NvS segment. The physical size of this translocation spans 16 cM, which corresponds to approximately 32.6–33.5 Mb of the total 2A wheat chromosome [61]. This translocation not only provides resistance to multiple pathogens [62] but also plays a role in increasing wheat yield [61]. This translocation has been frequently present in CIMMYT germplasm [34,63]. The size estimation of 2NS/2AS translocation supports the identification of *QLrYr.cim-2AS* as *Yr17*. Additionally, two STS markers and one KASP marker closely linked to *Yr17* were used for the validation, further confirming *QLrYr.cim-2AS* to be the same locus. Despite the documented global virulence of *Yr17*, the prevalent *Pst* race (MEX14.191) in Mexico is likely to exhibit a partial virulence on *Yr17*, resulting in low IT resembling the *Yr17* IT of 23C in our bi-parental population. Although the standard YR differential near isogenic line (NIL) carrying *Yr17* in the Avocet background showed complete susceptibility in our study, this finding confirms that the expression of *Yr17* varies significantly due to the influence of genetic backgrounds and environmental conditions [34,64]. The Kenyan *Pst* races have complete virulence on *Yr17* [65], which explains why no 2AS QTL peak was observed with the Kenyan YR datasets.

For LR, no seedling resistance was observed and the *Pt* race, MBJ/SP used in this study is known for its virulence for *Lr37* [34,66]. In some instances, *Lr37* has been shown to provide quantitative resistance as the germplasm with this translocation exhibits optimal resistance in adult plants, whereas achieving low IT at seedling is often difficult [67,68]. Despite the documented virulence of *Lr37*, the QTL encompassing *Lr37* in our study unequivocally conferred resistance at the adult plant stage. This resistance effect may be attributed to an additional linked slow rusting gene within the translocated chromosomal segment from *Aegilops ventricosa* [34,63,69], or it goes along with previous arguments of *Lr37* providing quantitative resistance at plant stage.

The final dual co-located loci, *QLrYr.cim-3AL,* conferring resistance to both LR and YR, was identified on the telomeric region of chromosome 3AL, spanning approximately 747 Mb within the DArTSeq marker interval between *7334423* and *100098678*. A major QTL, *QLr.fcu-3AL*, conferring race-specific resistance at 709.4 Mb on chromosome 3AL from the synthetic hexaploid wheat line TA4152-60, was mapped [70]. Additionally, *QYr.hebau-3AL/QLr.hebau-3AL* was identified on chromosome 3AL within the 717.6–719.2 Mb marker interval, with a PVE of 4.9–8.9%, as reported by [71]. Another QTL, *QLr-3AL,* was detected through a GWAS study targeting LR resistance associated with the 90K Illumina iSelect SNP array marker, *RAC875_c65573_410 742.8,* mapped at 742.8 Mb [72]. Ref. [73] mapped a YR QTL, *QYr.rcrrc-3A,* spanning between 655.7 and 701.9 Mb on chromosome 3AL, with 3.68% PVE. Based on its physical proximity, *QLrYr.cim-3AL,* identified in this study, appears to overlap with *QLr-3AL*, suggesting potential similarity. In contrast, the other identified QTL on chromosome 3AL either have different modes of resistance or are located at different chromosomal positions.

### 3.2. Minor QTL Regions

#### 3.2.1. Genomic Regions Associated with LR Resistance

*QLr.cim-2DS* was a consistent and stable LR QTL across various environments and years in Mexico, located on the short arm of chromosome 2D, spanning approximately between 12 and 17 Mb on the IWGSC_RefSeq_v1.0 assembly. The majority of the genes reported on 2DS belong to the ASR category [74,75]. A leaf rust QTL, *QLr.cim-2DS*, corresponding to the 14–16 cM region on the consensus map, was reported by [34], aligning with the QTL identified in the current study. Additionally, *QLr.cim-2DS*, contributed by UC1110, was also reported in another previous study and appears to represent the same genetic locus based on marker position [76]. To further confirm the validity of *QLr.cim-2DS* as the same as previous studies, the marker *CIM0004*, linked to 2DS QTL [34], was genotyped in the current population. *CIM0004* was mapped among the linked DArTSeq markers, validating its association with *QLr.cim-2DS*.

*QLr.cim-6DS* was another consistent and stable QTL for LR identified on chromosome 6DS with 5–9% of the PVE. Not many LR genes and/or QTL have been reported on chromosome 6DS. Previously, *QLr.hebau-6DS* was detected within the marker interval of 39.5 and 41.5 Mb of IWGSC RefSeq v1.0 in an F6 RIL population [77]. The position of the linked markers indicates that *QLr.cim-6DS* is distinct from *QLr.hebau-6DS*, suggesting that *QLr.cim-6DS* may represent a novel QTL. These identified QTL provide valuable targets for marker-assisted selection (MAS) in breeding programs. Their stability across multiple environments suggests their potential for enhancing durable LR resistance in wheat, making them important candidates for introgression into elite germplasm.

#### 3.2.2. Genomic Regions Associated with YR Resistance

*QYrKen.cim-3DS* was identified only in Kenya at approximately 150 Mb on the short arm of chromosome 3D, conferring YR resistance with a PVE ranging from 6.3% to 7.8%. Numerous other genes and QTL associated with YR resistance have been reported on chromosome 3DS, including *QYr.ucw-3D* [78], *QYr.tam-3D* [79], *QYr.cim-3D* [80], *QYr.cim-3DC* [76], and *QYr.inra-3DS* [81]. These QTL are predominantly located near the centromeric region of chromosome 3DS, which is about 237–243.5 Mb of the total 641 Mb chromosome length [82], suggesting *QYr.cim-3DS* to be distinct. Additionally, several other genes and QTL are also mapped on 3DS, such as *Yr49* at 7.1 Mb [83], *Yr66* at 3.5 Mb [84], *YrY206* at 7.1 Mb [85], *YrS1* at 13.1 Mb based on the linked markers, *Xcfd79* and *Xwmc674* [86] (https://plantinformatics.io/: accessed on 21 August 2024), *QYrsn.nwafu-3DS* around 3.5 Mb [87], *QYr.jki-3D* between 19.8 Mb and 22.0 Mb [88], and *QYr.cim-3DS* spanning from 78.7 Mb to 90.7 Mb [63]. Notably, *YrS1*, *Yr66,* and *QYrsn.nwafu-3DS* provide seedling resistance and are located in different regions on 3DS compared to our QTL, whereas *Yr49*, *YrY206*, *QYr.jki-3D*, and *QYr.cim-3DS* represent APR loci and are distinctly positioned from the QTL identified in our study, underscoring the novelty of *QYrKen.cim-3DS*.

*QYrKen.cim-6BS* was located on the short arm of chromosome 6B harboring several genes and QTL, including *Yr35* [89], *Yr36* [90], and *Yr78* [91]. In the current study, *QYrKen.cim-6BS* was identified only with Kenya datasets and was mapped between markers *1116951* and *3941697*, spanning 87 to 120 Mb on the IWGSC_RefSeq_v1.0 assembly [33]. *Yr35* and *Yr36* were introduced from *Triticum turgidum* ssp. *dicoccoides* into wheat, with *Yr35* providing race-specific resistance and *Yr36* offering high-temperature adult-plant partial resistance. Given their distinct resistance mechanism and genetic sources, it is unlikely that both the genes represent the same locus as *QYr.cim-6BS*. Many previously reported QTL on 6BS, such as *QYrsn.nwafu-6BS* [87], *QYr.wgp-6BS.1* [92], *QYr.sun-6BS* [93], *QYr.cim-6BS* [63], and *QYrMa.wgp-6BS* [94], were likely to be *Yr78* based on various studies [87,91,94]. The marker *IWA7257*, linked to *Yr78,* was mapped on the 92.46 Mb on the IWGSC_RefSeq_v1.0 assembly [33] of chromosome 6BS. *QYr.sicau-6BS* [95], located approximately 7.6 Mb distal to *IWA7257*, was suggested to be distinct from *Yr78*. Genotyping of the parental line, Kasuku, by marker *IWA7257* produced the T:T allele, indicative of *Yr78* presence, which was followed by genotyping in the RIL population. Integration of this genotypic data into linkage map confirmed the presence of the *IWA7257* marker within the interval of our identified QTL, suggesting that *QYrKen.cim-6BS* could correspond to *Yr78*.

#### 3.2.3. Genomic Regions Associated with SR Resistance

*QSr.cim-2BS* was another QTL identified on chromosome 2BS within the marker interval of *1026962* and *3021198,* located at around 68–76 Mb on the IWGSC_RefSeq_v1.0 assembly [33]. Chromosome 2BS harbors numerous genes and QTL associated with SR resistance, including *Sr36* from *Triticum timopheevi* [74], *Sr39* from *Aegilops speltoides* [96], and *Sr40* also from *Triticum timopheevi* [97], as well as *QSr.cdl-2BS.2* [98], *QSr.umn-2B.2* [99], *QSr.cdl-2BS* [100], *QSr.ufs-2B* [101], and *QSr.rwg-2B.1* [102]. *Sr36*, an ASR gene, does not confer resistance against race TTTSK race [103], while *Sr39* and *Sr40*, also ASR genes, effectively combat current prevalent races but are less frequent in modern germplasm due to potential linkage drag. Our study identifies a minor QTL explaining 7–9.3% of phenotypic variance, suggesting its distinction from *Sr36*, *Sr39,* and *Sr40*. Notably, none of Kasuku’s parentage apparently carry the *T. timopheevi* translocation, further distinguishing *QSr.cim-2BS*. Additional QTL include *QSr.umn-2B.2* (mapped at 22.7–40.7 Mb), *QSr.cdl-2BS* (12–31.7 Mb), *QSr.ufs-2B* (100.8 Mb), *QSr.cdl-2BS.2* (58.3–64.98 Mb), and *QSr.rwg-2B.1* (mapped at two intervals; 52.98–63.2 Mb and 96.2 Mb). Based on physical positioning, *QSr.umn-2B.2*, *QSr.cdl-2BS*, and *QSr.ufs-2B* likely represent different loci. Although *QSr.rwg-2B.1* and *QSr.cdl-2BS.2* overlap with our QTL region, the proportion of PVE by *QSr.umn-2B.2* (10.5–17.6%) and *QSr.cdl-2BS.2* (33.3%) significantly exceed our QTL, indicating probable differentiation. Further investigations are necessary to validate these findings.

*QSr.cim-5AL* was mapped on the telomeric region of the long arm of chromosome 5A, spanning approximately 682 Mb, with about 7% of PVE. Notably, no SR gene has been documented on chromosome 5A, and only a limited number of QTL have been identified. *QSr.cim-5A*, identified in an Avocet/Pavon76 RIL population, accounted for 6.3% of PVE for SR resistance, mapped within the marker interval of *XwPt-6048* and *XwPt-4249* [104,105]. Marker *XwPt-6048*, mapped at 59.5 cM on the consensus map by [106], corresponds to about 86 Mb on the IWGSC_RefSeq_v1.0_DArT_ver3_markers (https://plantinformatics.io/: accessed on 21 August 2024), indicating its location on the short arm of chromosome 5A. Additionally, through association mapping studies, two QTL have been identified: one linked to marker *gwm126* at 671.3 Mb and another linked to marker *gwm291* at 698.1 Mb on the IWGSC_RefSeq_v1.0, with 4.1% and 4.4% of PVE, respectively [107]. Another race-specific QTL, *5A.3* was characterized on chromosome 5AL [108], associated with marker *wsnp_Ku_c18023_27232712,* spanning 630 Mb on *Triticum turgidum*, Svevo assembly [109], corresponding to 672.4 Mb on IWGSC_RefSeq_v1.0 [33]. They also reported another QTL, *5A.2*, conferring APR with 10.8% of PVE, linked to marker *CAP7_c4064_162* at 649.3 Mb on the Svevo assembly [108], corresponding to 689.9 Mb on the IWGSC_RefSeq_v1.0. Based on the physical locations of these linked markers, it suggests that the QTL identified in our study may correspond to the APR QTL detected in studies by [107,108]. Further research is necessary to confirm the identity of the QTL.

*QSr.cim-6AS* was mapped within the DArTSeq marker interval of *3025616* and *3222462,* spanning approximately 9–10 Mb on the short arm of chromosome 6A. Several genes and QTL associated with SR resistance have been documented on this chromosome arm. These include the *Sr8* locus and its alleles, *Sr8a* and *Sr8b* [110], *Sr8155B1*, a potential allele of *Sr8* located between 6.7–10.9 Mb [98], *QSr_CxN.6A.1* and *QSr_NCCR.6A.1,* mapped at the telomeric region between 6.2 and 7.4 Mb on the 6AS chromosome [60], and *QSr.cim-6AS, *mapped around 13 Mb [34]. *QSr_CxN.6A.1* and *QSr_NCCR.6A.1* are considered to correspond to the *Sr8155B1* region based on their physical location and effectiveness against TTKST and TRTTF races [60]. The majority of Ug99 races used in Njoro, Kenya, exhibit virulence towards the *Sr8a* locus [111], suggesting that *QSr.cim-6AS* identified in this study is distinct from *Sr8a*. Although markers linked to *Sr8155b* did not provide conclusive results, the resistance of Kasuku against the virulent pathotype of *Sr8155b* confirms the distinct nature of this QTL. QTL reported by [34], located around 13 Mb on the IWGSC_RefSeq_v1.0 assembly, may represent the same locus identified in our study, indicating that these QTL could potentially represent another allele of *Sr8*. While these findings provide valuable insights into rust resistance in Kasuku, extending these results to other wheat backgrounds may present challenges due to genetic background effects and genotype × environment (G × E) interactions, where resistance expression can vary depending on the specific combination of resistance genes present in a given cultivar. Further validation in diverse wheat germplasm is necessary to confirm the consistency of these QTL across different genetic backgrounds. However, transferring these QTL into other CIMMYT germplasms may not be a major challenge, as it consists of elite breeding lines with high genetic similarity, facilitating the effective introgression of resistance loci.

Additionally, the regional specificity of pathogen virulence profiles complicates the widespread deployment of these resistance loci, as certain QTL may be more effective in some environments than others. However, the extension of this study to East Africa allowed the identification of new QTL under diverse virulence conditions, providing broader applicability. Notably, South Asian YR virulence profiles closely resemble those in East Africa, making these QTL highly relevant for CIMMYT breeding programs aimed at deployment in South Asia. Similarly, for SR, the identified QTL target resistance against Ug99 and its derivatives which are not yet widespread. This makes their integration into breeding programs a preemptive strategy to mitigate potential future incursions of these highly virulent races.

## 4. Materials and Methods

### 4.1. Plant and Pathogen Materials

“Kasuku”, a triple rust resistant wheat variety released in Kenya, was developed using the old Kenyan variety “Kenya Swara”, known to carry resistance to Ug99 races of SR, and Borlaug100 F2014, a variety released in Mexico combining high yield and YR/LR resistance. Kasuku was used to generate a RIL population by crossing it with triple rust-susceptible RIL selection, Apav#1, to form a bi-parental population comprising 181 lines. A differential set of 51 near-isogenic lines (NILs) in Avocet background for YR and 51 NILs in Thatcher background for LR alongside standard checks were also phenotyped with RIL population.

Predominantly prevalent Mexican *Pst* race MEX14.191, having avirulence/virulence: *Yr1*, *4*, *5a*, *10*, *15*, (*17*), *24*, *26*, *5b*, *Poll*/*Yr2*, *3*, *6*, *7*, *8*, *9*, *27*, *31*, *A* [77], was used for YR, while the predominantly prevalent *Pt* race, MBJ/SP, having avirulence/virulence: *Lr2a*, *2b*, *2c*, *3ka*, *9*, *16*, *19*, *21*, *24*, *25*, *28*, *29*, *30*, *32*, *33*, *36*/*1*, *3*, *3bg*, *10*, *11*, *12*, *13*, *14a*, *14b*, *15*, *17a*, *18*, *20*, *23*, (*26*), *27 + 31*, *37* [66], was used for LR in Mexico for both greenhouse and field experiments.

A mixture of predominantly prevalent *Pgt* races with various virulences [112], such as TTKSK (avirulence/virulence: *Sr24*, *36*, *Tmp*/*Sr5*, *6*, *7b*, *8a*, *9a*, *9b*, *9d*, *9e*, *9g*, *10*, *11*, *17*, *30*, *31*, *38*, *McN*), TTKST (avirulence/virulence: *Sr9h*, *36*, *Tmp*/*5*, *6*, *7b*, *8a*, *9a*, *9d*, *9g*, *9b*, *9e*, *10*, *11*, *17*, *21, 24*, *30*, *31*, *38*, *McN*), TTKTK (avirulence/virulence:, *Sr9h*, *24*, *36*/5, *21*, *9e*, *7b*, *11*, *6*, *8a*, *9g*, *9b*, *30*, *17*, *9a*, *9d*, *10*, *Tmp*, *31*, *38*, *McN*), and TTKTT (avirulence/virulence: *Sr9h*, *36*/*5*, *21*, *9e*, *7b*, *11*, *6*, *8a*, *9g*, *9b*, *30*, *17*, *9a*, *9d*, *10*, *24*, *31*, *38*, McN, *Tmp*) were used for SR phenotyping at KALRO research station in Njoro, Kenya, while YR resistance was evaluated under natural conditions.

### 4.2. Greenhouse Screening

Seedling phenotyping was carried out in El-Batan, Mexico, by testing the RIL population against stripe rust and leaf rust using MEX14.191 and MBJ/SP pathotypes, respectively. About 30 cm long and 23 cm wide trays were used for sowing, with 30 lines per tray and 5–6 seeds per line. The distance of 4 cm was kept between each RIL. After sowing, the trays were kept in a rust-free microclimate room at 20 °C under natural light conditions. Seedlings were inoculated with *Pst* and *Pt* pathotypes at the two-leaf stage. Urediniospores were suspended in the light mineral oil Soltrol-170 and were atomized on the seedlings. Post inoculations, stripe rust inoculated seedlings were transferred to a due chamber at 7–9 °C for 24 h while leaf rust inoculated seedlings were moved to the humidified chamber for 24 h. After 24 h, stripe rust and leaf rust inoculated seedlings were then transferred to microclimate rooms set at 17 ± 2 and 25 ± 2 °C, respectively, with more than 80% humidity. Rust assessment was made after 12–14 days of inoculation using the 0–4 scale described in [74]. IT 0 to 3 were considered resistant, and 3^+^ and 4 were considered susceptible, where IT 0 = no visible infection/immune response, IT ; = necrotic flecks with no uredinia, IT 1 = small uredinia with necrosis and/or chlorosis, IT 2 = small to medium uredinia with characteristic chlorotic, necrotic border, IT 3 = medium-sized uredinia chlorosis, and IT 4 = larger uredinia with profuse sporulation and no necrosis/chlorosis. C and N were used to indicate more than usual degrees of chlorosis and necrosis, respectively.

### 4.3. Field Screening

Field trials for LR and YR were conducted in Mexico, while SR and YR were evaluated at KALRO in Njoro, Kenya, at the phenotyping platforms specifically developed as rust hotspots with a conducive environment for disease development. The RIL population was sown in 0.7 m rows with a 0.3 m path in between each row. The whole trial was surrounded by a spreader row, which consisted of susceptible genotypes/cultivars both in Mexico and Kenya for uniform disease development. The spreader rows were sown as hill plots in between the trial rows.

For LR, the entire RIL population was tested for the years 2019 and 2022 at the CIMMYT experimental station in Obregon, Mexico (latitude 27.33, longitude −109.93, 39 masl), and for the years 2020 and 2021 at the CIMMYT headquarters in El-Batan, Mexico (latitude 19.528192, longitude −98.84794, 2249 masl). YR evaluations were carried out for two years in 2021 and 2022 at the CIMMYT station in Toluca, Mexico (latitude 19.226581, longitude −99.551539, 2640 masl). Testing for YR and SR in Kenya was carried out at KALRO, Njoro, Kenya (latitude −0.341368, longitude 35.947650, 2165 masl), for the years 2021 and 2022. Predominant pathotypes were used for inoculating the spreader rows, which were a mix of universal susceptible varieties in both Mexico and Kenya to create an artificial epidemic, as mentioned in [34].

The percent disease severity of infected flag leaves was scored using the modified Cobb disease scale of 0–100 [113] at weekly intervals at all locations while the infection type assessment was recorded as resistant (R)—small uredinia with chlorosis or necrosis; moderately resistant (MR)—medium-sized uredinia with necrosis or chlorosis; moderately susceptible (MS)—medium-sized uredinia with or without some chlorosis; susceptible (S)—large uredinia without chlorosis and necrosis [114]. The first dataset was recorded when the susceptible parental line reached 80% disease severity. Disease evaluations were conducted two to three times at 7–8-day intervals following the initial scoring. The sequential assessments were labeled as follows: “1” (first dataset), “2” (second dataset, recorded 7–8 days after the first), and “3” (third dataset, recorded 14–16 days after the first).

Dataset designations followed a structured naming convention, where LR, YR, and SR represent the disease type (LR = leaf rust, YR = yellow rust, SR = stem rust), the two-digit year indicates the year of evaluation (19 = 2019, 20 = 2020, 21 = 2021, etc.), and the final digit denotes the sequential dataset number indicating repeated disease scoring within a given season. For example, LR19-1 refers to the first leaf rust dataset in 2019, SR21-2 represents the second stem rust dataset in 2021, and YRKEN23-3 corresponds to the third yellow rust dataset recorded in 2023 in Kenya. This systematic naming pattern was applied consistently across all datasets to ensure clarity and prevent ambiguity.

### 4.4. Statistical Analysis

A chi-squared analysis was performed to determine the goodness of fit of the observed segregation to the expected genetic ratios of the RIL population. Pearson’s correlation coefficients between mean disease severities were calculated by IBM SPSS Statistics 21.0 (IBM Corp., Armonk, NY, USA). To compare the mean disease severity of the RILs carrying various combinations of QTLs, the least significant difference (LSD) was calculated.

### 4.5. Genotyping and Linkage Map Construction

Genomic DNA was extracted from 10-day-old seedlings of the entire RIL population, along with parents, using a modified cetyltrimethylammonium bromide (CTAB) method described by [115]. DNA quality was assessed using a Nanodrop 8000 spectrophotometer (Thermo Scientific, Waltham, MA, USA), and samples were diluted to 200 ng/μL. The entire RIL population and parental lines were genotyped using the DArTSeq platform at the Genetic Analysis Service for Agriculture (SAGA) in CIMMYT’s Biotech Laboratory (https://seedsofdiscovery.org/about/genotyping-platform/: accessed on 30 May 2023).

The DArTSeq markers were quality filtered by removing parental non-polymorphic markers, markers with more than 20% missing data, and markers with greater than 20% heterozygous calls. Goodness of fit between observed and expected segregation ratios was observed using the chi-squared test. Markers showing segregation distortion (χ^2^ > 5) were excluded. The markers were grouped using the regression method at a minimum LOD threshold of 5 using the ASMap package in R software V-4.0.4 according to the protocol mentioned in [116,117]. Recombination frequencies were converted into map distances (centiMorgans) using the Kosambi mapping function. Marker positions within each linkage group were determined by assessing their contributions to the average goodness of fit, i.e., Chi-square and the nearest neighbor fit values. Markers were added or removed based on these criteria, and calculations were repeated until the optimal fit and order were achieved.

### 4.6. QTL Analysis

The final linkage map for QTL analysis was prepared by using the final genetic map and phenotypic data through MapManager QTXb20 [118]. QTL mapping was performed using QTL Cartographer v2.5 [119] based on composite interval mapping with 1000 permutations and minimum LOD score of 3. Stepwise regression was used to estimate the explained PVE percentage to identify significant QTL. MapChart v2.3 software was used to draw the genetic linkage maps [120]. QTL were named with *Q* (for QTL), name of the trait (Lr for leaf rust, Yr for yellow rust, and Sr for stem rust), followed by name of institute (cim = CIMMYT), and chromosome number and arm where the QTL lies, e.g., *QLrcim-2DS.*

### 4.7. Evaluation of Marker Selectable Resistance Genes

Gene-specific markers were selected based on identified QTL regions where previously reported resistance genes were known to be present. Using published information, linked markers were mapped and incorporated into the linkage maps to validate their association with known loci. Gene-specific markers for *Lr46*/*Yr29*/*Sr58*; Kompetitive allele-specific polymerase chain reaction (KASP) marker *SNPG122*, *Yr17*/*Lr37*/*Sr38*; one KASP marker, *Yr17*/*Lr37*/*Sr58* [121], and two sequence tagged site (STS) markers, *WGGB156* [117] and *cslVrgal3* [118]; for 2D QTL, *CIM0004* and *IWA7257* for *Yr78* were tested for the presence of these genes. For KASP markers, the assay was performed according to [4], and for STS markers, their respective protocols were followed according to [122,123].

## 5. Conclusions

In conclusion, the ongoing challenge posed by rust pathogens necessitates a strategic approach to enhancing wheat rust resistance through the identification and characterization of durable resistance loci. This study successfully identified significant genomic regions associated with APR to multiple rust diseases, particularly through the Kenyan variety “Kasuku.” The discovery of co-located loci such as *QLrYrSr.cim-1BL*, *QLrYr.cim-2AS*, *QLrYrSr.cim-3AL,* and *QLrYrSr.cim-6AL*, which exhibit broad-spectrum resistance, highlights their potential role in CIMMYT’s breeding programs aimed at developing resilient wheat cultivars.

The consistent expression of these QTL across different environments further underscores their importance in breeding strategies, as they provide a stable foundation for combating the evolving rust threats. By integrating these findings into wheat breeding, we can advance the development of germplasm that not only enhances yield and adaptability but also fortifies resistance against biotic stresses. Continued research and validation of these loci will be essential for harnessing their full potential, ensuring sustainable wheat production in the face of global agricultural challenges.

## Figures and Tables

**Table 1:** Pearson correlation coefficient (r) for two-way comparisons of leaf rust and stripe rust disease severity data from different environments in Mexico.

	LR19-1	LR19-2	LR20	LR21-1	LR21-2	LR22-1	LR22-2	YR20-1	YR20-2	YR21-1	YR21-2
LR19-1	1.00							-	-	-	-
LR19-2	0.81	1.00						-	-	-	-
LR20	0.76	0.85	1.00					-	-	-	-
LR21-1	0.78	0.84	0.79	1.00				-	-	-	-
LR21-2	0.78	0.81	0.81	0.91	1.00			-	-	-	-
LR22-1	0.76	0.76	0.77	0.75	0.71	1.00		-	-	-	-
LR22-2	0.80	0.85	0.86	0.81	0.78	0.94	1.00	-	-	-	-
YR20-1	-	-	-	-	-	-	-	1.00			
YR20-2	-	-	-	-	-	-	-	0.91	1.00		
YR21-1	-	-	-	-	-	-	-	0.88	0.87	1.00	
YR21-2	-	-	-	-	-	-	-	0.86	0.85	0.96	1.00

All correlations are significant at *p* < 0.01. LR indicates leaf rust; YR indicates stripe rust in Mexico. The numbers 19, 20, 21, and 22 indicate the years 2019, 2020, 2021, and 2022, respectively. After the year, numbers 1 and 2 correspond to the first and second datasets, respectively, each collected at one-week interval.

**Table 2:** Pearson correlation coefficient (r) for two-way comparisons of stripe rust and stem rust disease severity data from different environments in Kenya.

	YRKEN21-1	YRKEN21-2	YRKEN21-3	YRKEN23-1	YRKEN23-2	SR21-1	SR21-2	SR21-3	SR23-1	SR23-2
YRKEN21-1	1.00					-	-	-	-	-
YRKEN21-2	0.78	1.00				-	-	-	-	-
YRKEN21-3	0.75	0.94	1.00			-	-	-	-	-
YRKEN23-1	0.75	0.77	0.70	1.00		-	-	-	-	-
YRKEN23-2	0.76	0.75	0.75	0.85	1.00	-	-	-	-	-
SR21-1	-	-	-	-	-	1.00				
SR21-2	-	-	-	-	-	0.94	1.00			
SR21-3	-	-	-	-	-	0.88	0.94	1.00		
SR23-1	-	-	-	-	-	0.70	0.74	0.72	1.00	
SR23-2	-	-	-	-	-	0.72	0.75	0.75	0.95	1.00

All correlations are significant at *p* < 0.01. YRKEN indicates stripe rust in Kenya; SR indicates stem rust. The numbers 21 and 23 indicate the years 2021 and 2023, respectively. After the year, numbers 1 and 2 correspond to the first and second datasets, respectively, each collected at one-week interval.

**Table 3:** Summary of identified genetic loci for stripe rust, leaf rust, and stem rust in the Kasuku/Apav#1 recombinant inbred line (RIL) population.

QTL	Years QTL Identified	Environment	Chromosomal Location	QTL Associations with Rust Diseases
*QLrYrSr.cim-1BL*	2019, 2020, 2021, 2022	Obregon, El-Batan, Toluca, Njoro	1BL (678–685 Mb)	Co-located QTL for LR, YR, and SR
*QLrYr.cim-2AS*	2019, 2020, 2021, 2022	Obregon, El-Batan, Toluca	2AS (~8 Mb)	Co-located QTL for LR and YR
*QLrYr.cim-3AL*	2019, 2020, 2021, 2022	Obregon, El-Batan, Toluca	3AL (~747 Mb)	Co-located QTL for LR and YR
*QLrYrSr.cim-6AL*	2019, 2020, 2021, 2022, 2023	Obregon, El-Batan, Toluca, Njoro	6AL (609–614 Mb)	Co-located QTL for LR, YR, and SR
*QLr.cim-2DS*	2019, 2020, 2021, 2022	Obregon, El-Batan	2DS (12–17 Mb)	QTL for LR
*QLr.cim-6DS*	2019, 2020, 2021, 2022	Obregon, El-Batan	6DS (7–19 Mb)	QTL for LR
*QYrKen.cim-3DS*	2021, 2023	Njoro	3DS (~150 Mb)	QTL for YR
*QYrKen.cim-6BS*	2021, 2023	Njoro	6BS (87–120 Mb)	QTL for YR
*QSr.cim-2BS*	2021, 2023	Njoro	2BS (68–76 Mb)	QTL for SR
*QSr.cim-5AL*	2021, 2023	Njoro	5AL (682 Mb)	QTL for SR
*QSr.cim-6AS*	2021, 2023	Njoro	6AS (9–10 Mb)	QTL for SR

## Data Availability

The data used to present the findings reported in this study is available upon request through the corresponding author. The data are not publicly available due to [storage limitations of large size of genotypic files].

## Supplementary Materials

The following supporting information can be downloaded at: https://www.mdpi.com/article/10.3390/plants14071007/s1, Table S1: Summary of Identified Genetic Loci for Stripe Rust, Leaf Rust, and Stem Rust in the Kasuku/Apav#1 Recombinant Inbred Line (RIL) Population, including LOD Scores, Phenotypic Variance Explained (R2), and Physical Positions (Mb).

## References

[1] Dean R., Van Kan J.A.L., Pretorius Z.A., Hammond-Kosack K.E., Di Pietro A., Spanu P.D., Rudd J.J., Dickman M., Kahmann R., Ellis J. (2012). The Top 10 fungal pathogens in molecular plant pathology. Mol. Plant Pathol..

[2] Saari E.E., Prescott J.M., Roelfs A.P., Bushnell W.R. (1985). World distribution in relation to economic losses. The Cereal Rusts Volume II: Diseases, Distribution, Epidemiology and Control.

[3] Figueroa M., Hammond-Kosack K.E., Solomon P.S. (2018). A review of wheat diseases-a field perspective. Mol. Plant Pathol..

[4] Qureshi N. (2017). Rust Resistance in Wheat: Gene Discovery and Development of Molecular Markers Using Diverse Genomic Resources. Ph.D. Thesis.

[5] Oliver R.P. (2014). A reassessment of the risk of rust fungi developing resistance to fungicides. Pest Manag. Sci..

[6] Bariana H.S., Thomas B., Murphy D.J., Murray B.G. (2003). Breeding for Disease Resistance. Encyclopedia of Applied Plant Sciences.

[7] Chen X.M. (2005). Epidemiology and control of stripe rust [*Puccinia striiformis* f. sp. *tritici*] on wheat. Can. J. Plant Pathol..

[8] Ellis J.G., Lagudah E.S., Spielmeyer W., Dodds P.N. (2014). The past, present and future of breeding rust resistant wheat. Front. Plant Sci..

[9] Kolmer J.A. (1996). Genetics of resistance to wheat leaf rust. Annu. Rev. Phytopathol..

[10] Sucher J., Boni R., Yang P., Rogowsky P., Büchner H., Kastner C., Kumlehn J., Krattinger S.G., Keller B. (2017). The durable wheat disease resistance gene Lr34 confers common rust and northern corn leaf blight resistance in maize. Plant Biotechnol. J..

[11] Agrios G. (2005). Plant Pathology.

[12] Kolmer J. (2013). Leaf Rust of Wheat: Pathogen Biology, Variation and Host Resistance. Forests.

[13] Brunner S., Stirnweis D., Diaz Quijano C., Buesing G., Herren G., Parlange F., Barret P., Tassy C., Sautter C., Winzeler M. (2012). Transgenic *Pm3* multilines of wheat show increased powdery mildew resistance in the field. Plant Biotechnol. J..

[14] Dyck P.L., Samborsk D.J., Anderson R.G. (1966). Inheritance of adult-plant leaf rust resistance derived from common wheat varieties Exchange and Frontana. Can. J. Genet. Cytol..

[15] Singh R.P. (1992). Genetic association of leaf rust resistance gene Lr34 with adult plant resistance to stripe rust in bread wheat. Phytopathol.

[16] Spielmeyer W., McIntosh R.A., Kolmer J., Lagudah E.S. (2005). Powdery mildew resistance and *Lr34/Yr18* genes for durable resistance to leaf and stripe rust cosegregate at a locus on the short arm of chromosome 7D of wheat. Theor. Appl. Genet..

[17] Lillemo M., Asalf B., Singh R.P., Huerta-Espino J., Chen X.M., He Z.H., Bjørnstad A. (2008). The adult plant rust resistance loci *Lr34/Yr18* and *Lr46/Yr29* are important determinants of partial resistance to powdery mildew in bread wheat line Saar. Theor. Appl. Genet..

[18] Singh R., Herrera-Foessel S.A., Huerta-Espino J., Bariana H.S., Bansal U., McCallum B.D., Hiebert C., Bhavani S., Singh S., Lan C.X. (2012). *Lr34/Yr18/Sr57/Pm38/Bdv1/Ltn1* confers slow rusting, adult plant resistance to *Puccinia graminis* f. sp. *tritici*. Proceedings of the 13th Cereal Rust and Powdery Mildew Conference.

[19] Lillemo M., Joshi A.K., Prasad R., Chand R., Singh R.P. (2013). QTL for spot blotch in bread wheat line Saar co-locate to the biotrophic disease resistance loci *Lr34* and *Lr46*. Theor. Appl. Genet..

[20] Singh R.P., Herrera-Foessel S.A., Huerta-Espino J., Lan C., Basnet B.R., Bhavani S., Lagudah E.S. Pleiotropic gene *Lr46/Yr29/Pm39/Ltn2* confers slow rusting.; adult plant resistance to wheat stem rust fungus. Proceedings of the Borlaug Global Rust Initiative, 2013 Technical Workshop.

[21] Herrera-Foessel S.A., Singh R.P., Lillemo M., Huerta-Espino J., Bhavani S., Singh S., Lan C., Calvo-Salazar V., Lagudah E.S. (2014). *Lr67/Yr46* confers adult plant resistance to stem rust and powdery mildew in wheat. Theor. Appl. Genet..

[22] Dyck P.L. (1991). Genetics of adult-plant leaf rust resistance in ‘Chinese spring’ and ‘sturdy’ wheats. Crop Sci..

[23] Lagudah E.S., McFadden H., Singh R.P., Huerta-Espino J., Bariana H.S., Spielmeyer W. (2006). Molecular genetic characterization of the *Lr34⁄Yr18* slow rusting resistance gene region in wheat. Theor. Appl. Genet..

[24] Shah S.J.A., Hussain S., Ahmad M., Farhatullah A.I., Ibrahim M. (2011). Using leaf tip necrosis as a phenotypic marker to predict the presence of durable rust resistance gene pair *Lr34/Yr18* in wheat. J. Gen. Plant Pathol..

[25] Singh R.P. (1992). Association between gene *Lr34* for leaf rust resistance and leaf tip necrosis in wheat. Crop Sci..

[26] McFadden E.S. (1930). A successful transfer of emmer characters to vulgare wheat. J. Am. Soc. Agron..

[27] Singh R.P., Huerta-Espino J., Rajaram S. (2000). Achieving near-immunity to leaf and stripe rusts in wheat by combining slow rusting resistance genes. Acta Phytopathol. Entomol. Hung..

[28] da Silva G.B.P., Zanella C.M., Martinelli J.A., Chaves M.S., Hiebert C.W., McCallum B.D., Boyd L.A. (2018). Quantitative trait loci conferring leaf rust resistance in hexaploid wheat. Phytopathology.

[29] Elshire R.J., Glaubitz J.C., Sun Q., Poland J.A., Kawamoto K., Buckler E.S., Mitchell S.E. (2011). A robust, simple genotyping-by-sequencing (GBS) approach for high diversity species. PLoS ONE.

[30] Cavanagh C.R., Chao S., Wang S., Huang B.E., Stephen S., Kiani S., Forrest K., Saintenac C., Brown-Guedira G.L., Akhunova A. (2013). Genome-wide comparative diversity uncovers multiple targets of selection for improvement in hexaploid wheat landraces and cultivars. Proc. Natl. Acad. Sci. USA.

[31] Wang S., Wong D., Forrest K., Allen A., Chao S., Huang B.E., Maccaferri M., Salvi S., Milner S.G., Cattivelli L. (2014). Characterization of polyploid wheat genomic diversity using a high-density 90,000 single nucleotide polymorphism array. Plant Biotechnol. J..

[32] Akbari M., Wenzl P., Caig V., Carling J., Xia L., Yang S., Uszynski G., Mohler V., Lehmensiek A., Kuchel H. (2006). Diversity arrays technology (DArT) for high-throughput profiling of the hexaploid wheat genome. Theor. Appl. Genet..

[33] International Wheat Genome Sequencing Consortium (IWGSC) (2018). Shifting the limits in wheat research and breeding using a fully annotated reference genome. Science.

[34] Qureshi N., Singh R.P., Gonzalez B.M., Velazquez-Miranda H., Bhavani S. (2023). Genomic regions associated with resistance to three rusts in CIMMYT wheat line “Mokue#1”. Int. J. Mol. Sci..

[35] Singh R.P., Huerta-Espino J., Bhavani S., Herrera-Foessel S.A., Singh D., Singh P.K., Velu G., Mason R.E., Jin Y., Njau P. (2011). Race non-specific resistance to rust diseases in CIMMYT spring wheats. Euphytica.

[36] Kolmer J.A. (2015). A QTL on chromosome 5BL in wheat enhances leaf rust resistance of *Lr46*. Mol. Breed..

[37] Rosewarne G.M., Herrera-Foessel S.A., Singh R.P., Huerta-Espino J., Lan C.X., He Z.H. (2013). Quantitative trait loci of stripe rust resistance in wheat. Theor. Appl. Genet..

[38] Rosewarne G.M., Singh R.P., Huerta-Espino J., William H.M., Bouchet S., Cloutier S., McFadden H., Lagudah E.S. (2006). Leaf tip necrosis, molecular markers and beta1-proteasome subunits associated with the slow rusting resistance genes *Lr46/Yr29*. Theor. Appl. Genet..

[39] Marais G.F., Badenhorst P.E., Eksteen A., Pretorius Z.A. (2010). Reduction of *Aegilops sharonensis* chromatin associated with resistance genes *Lr56* and *Yr38* in wheat. Euphytica.

[40] Marais F., Marais A., McCallum B., Pretorius Z. (2009). Transfer of leaf rust and stripe rust resistance genes *Lr62* and *Yr42* from *Aegilops neglecta* Req. ex Bertol. to common wheat. Crop Sci..

[41] Cao J., Deng Z.Y., Wang M.N., Wang X.P., Jing J.X., Zhang X.Q., Shang H.S., Li Z.Q. (2008). Inheritance and molecular mapping of an alien stripe-rust resistance gene from a wheat-*Psathyrostachys huashanica* translocation line. Plant Sci..

[42] Kolmer J., Bernardo A., Bai G., Hayden M., Anderson J. (2019). Thatcher wheat line RL6149 carries *Lr64* and a second leaf rust resistance gene on chromosome 1DS. Theor. Appl. Genet..

[43] Ren Y., Singh R.P., Basnet B.R., Lan C.X., Huerta-Espino J., Lagudah E.S., Ponce-Molina L.J. (2017). Identification and mapping of adult plant resistance loci to leaf rust and stripe rust in common wheat cultivar Kundan. Plant Dis..

[44] Beukert U., Liu G., Thorwarth P., Boeven P.H.G., Longin C.F.H., Zhao Y., Ganal M., Serfling A., Ordon F., Reif J.C. (2020). The potential of hybrid breeding to enhance leaf rust and stripe rust resistance in wheat. Theor Appl Genet..

[45] Lin M., Dieseth J.A., Alsheikh M., Yang E., Holzapfel J., Schürmann F., Morales L., Michel S., Buerstmayr H., Bhavani S. (2023). A major yellow rust resistance QTL on chromosome 6A shows increased frequency in recent Norwegian spring wheat cultivars and breeding lines. Theor. Appl. Genet..

[46] Shahinnia F., Geyer M., Schürmann F., Rudolphi S., Holzapfel J., Kempf H., Stadlmeier M., Löschenberger F., Morales L., Buerstmayr H. (2022). Genome-wide association study and genomic prediction of resistance to stripe rust in current Central and Northern European winter wheat germplasm. Theor. Appl. Genet..

[47] Kale S.M., Schulthess A.W., Padmarasu S., Boeven P.H.G., Schacht J., Himmelbach A., Steuernagel B., Wulff B.B.H., Reif J.C., Stein N. (2022). Catalogue of resistance gene homologs and a chromosome-scale reference sequence support resistance gene mapping in winter wheat. Plant Biotechnol. J..

[48] Bouvet L., Percival-Alwyn L., Berry S., Fenwick P., Mantello C.C., Sharma R., Holdgate S., Mackay I.J., Cockram J. (2022). Wheat genetic loci conferring resistance to stripe rust in the face of genetically diverse races of the fungus *Puccinia striiformis* f. sp. *tritici*. Theor. Appl. Genet..

[49] Cheng B., Gao X., Cao N., Ding Y., Chen T., Zhou Q., Gao Y., Xin Z., Zhang L. (2022). QTL mapping for adult plant resistance to wheat stripe rust in M96-5 × Guixie 3 wheat population. J. Appl. Genet..

[50] Feng J., Chen G., Wei Y., Liu Y., Jiang Q., Li W., Pu Z., Lan X., Dai S., Zheng Y. (2014). Identification and genetic mapping of a recessive gene for resistance to stripe rust in wheat line LM168-1. Mol. Breed..

[51] Bulli P., Zhang J., Chao S., Chen X., Pumphrey M. (2016). Genetic architecture of resistance to stripe rust in a global winter wheat germplasm collection. G3 Genes Genomes Genet..

[52] Dolores Vazquez M., James Peterson C., Riera-Lizarazu O., Chen X., Heesacker A., Ammar K., Crossa J., Mundt C.C. (2012). Genetic analysis of adult plant.; quantitative resistance to stripe rust in wheat cultivar “Stephens” in multi-environment trials. Theor. Appl. Genet..

[53] William H.M., Singh R.P., Huerta-Espino J., Palacios G., Suenaga K. (2006). Characterization of genetic loci conferring adult plant resistance to leaf rust and stripe rust in spring wheat. Genome.

[54] Vazquez M.D., Zemetra R., Peterson C.J., Chen X.M., Heesacker A., Mundt C.C. (2015). Multi-location wheat stripe rust QTL analysis: Genetic background and epistatic interactions. Theor. Appl. Genet..

[55] Zhang L., Li Z., Lillemo M., Xia X., Liu D., Yang W., Luo J., Wang H. (2009). QTL mapping for adult-plant resistance to leaf rust in CIMMYT wheat cultivar Saar. Agric. Food Sci..

[56] Kumar D., Kumar A., Chhokar V., Gangwar O.P., Bhardwaj S.C., Sivasamy M., Prasad S.V.S., Prakasha T.L., Khan H., Singh R. (2020). Genome-wide association studies in diverse spring wheat panel for stripe, stem, and leaf rust resistance. Front. Plant Sci..

[57] Sharma J.S., Zhang Q., Rouse M.N., Klindworth D.L., Friesen T.L., Long Y., Olivera P.D., Jin Y., McClean P.E., Xu S.S. (2019). Mapping and characterization of two stem rust resistance genes derived from cultivated emmer wheat accession PI 193883. Theor. Appl. Genet..

[58] Sharma J.S., Che M., Fetch T., McCallum B.D., Xu S.S., Hiebert C.W. (2024). Identification of *Sr67*, a new gene for stem rust resistance in KU168-2 located close to the Sr13 locus in wheat. Theor. Appl. Genet..

[59] Megerssa S.H., Ammar K., Acevedo M., Brown-Guedira G., Ward B., Degete A.G., Randhawa M.S., Sorrells M.E. (2020). Multiple-race stem rust resistance loci identified in durum wheat using genome-wide association mapping. Front. Plant Sci..

[60] Marone D., Mazzucotelli E., Matny O., Desiderio F., Sciara G., Maccaferri M., Marcotuli I., Gadaleta A., Steffenson B., Mastrangelo A.M. (2022). QTL mapping of stem rust resistance in populations of durum wheat. Genes.

[61] Gao L., Koo D.H., Juliana P., Rife T., Singh D., Lemes da Silva C., Lux T., Dorn K.M., Clinesmith M., Silva P. (2021). The *Aegilops ventricosa* 2NvS segment in bread wheat: Cytology, genomics and breeding. Theor. Appl. Genet..

[62] Helguera M., Khan I.A., Kolmer J., Lijavetzky D., Zhong-Qi L., Dubcovsky J. (2003). PCR assays for the Lr37-Yr17-Sr38 cluster of rust resistance genes and their use to develop isogenic hard red spring wheat lines. Crop Sci..

[63] Liu D., Yuan C., Singh R.P., Randhawa M.S., Bhavani S., Kumar U., Huerta-Espino J., Lagudah E., Lan C. (2022). Stripe rust and leaf rust resistance in CIMMYT wheat line “Mucuy” is conferred by combinations of race-specific and adult-plant resistance loci. Front. Plant Sci..

[64] Sharma-Poudyal D., Chen X.M., Wan A.M., Zhan G.M., Kang Z.S., Cao S.Q., Jin S.L., Morgounov A., Akin B., Mert Z. (2013). Virulence characterization of international collections of the wheat stripe rust pathogen, *Puccinia striiformis* f. sp. *tritici*. Plant Dis..

[65] Hovmøller M.S. (2014). Report for Puccinia striiformis Race Analysis 2013, Global Rust Reference Center (GRRC), Aarhus University, Flakkebjerg, DK-4200 Slagelse, Denmark. https://agro.au.dk/fileadmin/Summary_of_Puccinia_striiformis_race_analyses_2013.pdf.

[66] Herrera-Foessel S.A., Singh R.P., Huerta-Espino J., Rosewarne G.M., Periyannan S.K., Viccars L., Calvo-Salazar V., Lan C., Lagudah E.S. (2012). Lr68: A new gene conferring slow rusting resistance to leaf rust in wheat. Theor. Appl. Genet..

[67] McIntosh R.A., Friebe B., Jiang J., The D., Gill B.S. (1995). Cytogenetical studies in wheat XVI. Chromosome location of a new gene for resistance to leaf rust in a Japanese wheat-rye translocation line. Euphytica.

[68] Xue S., Kolmer J.A., Wang S., Yan L. (2018). Mapping of leaf rust resistance genes and molecular characterization of the 2NS/2AS translocation in the wheat cultivar Jagger. G3 Genes Genomes Genet..

[69] Rauf Y., Lan C., Randhawa M., Singh R.P., Huerta-Espino J., Anderson J.A. (2022). Quantitative trait loci mapping reveals the complexity of adult plant resistance to leaf rust in spring wheat ‘Copio’. Crop Sci..

[70] Chu C.G., Friesen T.L., Xu S.S., Faris J.D., Kolmer J.A. (2009). Identification of novel QTLs for seedling and adult plant leaf rust resistance in a wheat doubled haploid population. Theor. Appl. Genet..

[71] Gebrewahid T.W., Zhang P., Zhou Y., Yan X., Xia X., He Z., Liu D., Li Z. (2020). QTL mapping of adult plant resistance to stripe rust and leaf rust in a Fuyu 3/Zhengzhou 5389 wheat population. Crop J..

[72] Zhang P., Yan X., Gebrewahid T.W., Zhou Y., Yang E., Xia X., He Z., Li Z., Liu D. (2021). Genome-wide association mapping of leaf rust and stripe rust resistance in wheat accessions using the 90K SNP array. Theor. Appl. Genet..

[73] Tehseen M.M., Tonk F.A., Tosun M., Randhawa H.S., Kurtulus E., Ozseven I., Akin B., Nur Zulfuagaoglu O., Nazari K. (2022). QTL Mapping of adult plant resistance to stripe rust in a doubled haploid wheat population. Front. Genet..

[74] McIntosh R.A., Welling C.R., Park R.F. (1995). Wheat Rusts, An Atlas of Resistance Genes.

[75] McIntosh R.A., Yamazaki Y., Dubcovsky J., Rogers J., Morris C., Somers D.J., Appels R., Devos K.M. (2008). Catalogue of Gene Symbols for Wheat, National BioResource Project, Komugi-Wheat Genetic Resources Database. http://shigen.nig.ac.jp/wheat/komugi/genes/download.jsp.

[76] Lan C., Hale I.L., Herrera-Foessel S.A., Basnet B.R., Randhawa M.S., Huerta-Espino J., Dubcovsky J., Singh R.P. (2017). Characterization and mapping of leaf rust and stripe rust resistance loci in hexaploid wheat lines UC1110 and PI610750 under Mexican environments. Front. Plant Sci..

[77] Zhang R., Singh R.P., Lillemo M., He X., Randhawa M.S., Huerta-Espino J., Singh P.K., Li Z., Lan C. (2019). Two main stripe rust resistance genes identified in synthetic-derived wheat line Soru# 1. Phytopathology.

[78] Cobo N., Pflüger L., Chen X.M., Dubcovsky J. (2018). Mapping QTL for resistance to new virulent races of wheat stripe rust from two Argentinean wheat cultivars. Crop Sci..

[79] Basnet B.R., Singh R.P., Ibrahim A.M.H., Herrera-Foessel S.A., Huerta-Espino J., Lan C., Rudd J.C. (2014). Characterization of *Yr54* and other genes associated with adult plant resistance to yellow rust and leaf rust in common wheat Quaiu 3. Mol. Breed..

[80] Yang E.N., Rosewarne G.M., Herrera-Foessel S.A., Huerta-Espino J., Tang Z.X., Sun C.F., Ren Z.L., Singh R.P. (2013). QTL analysis of the spring wheat “Chapio” identifies stable stripe rust resistance despite inter-continental genotype × environment interactions. Theor. Appl. Genet..

[81] Dedryver F., Paillard S., Mallard S., Robert O., Trottet M., Nègre S., Verplancke G., Jahier J. (2009). Characterization of genetic components involved in durable resistance to stripe rust in the bread wheat ‘Renan’. Phytopathology.

[82] Su H., Liu Y., Liu C., Shi Q., Huang Y., Han F. (2019). Centromere satellite repeats have undergone rapid changes in polyploid wheat subgenomes. Plant Cell.

[83] Somers D.J., Isaac P., Edwards K. (2004). A high-density microsatellite consensus map for bread wheat (*Triticum aestivum* L.). Theor. Appl. Genet..

[84] Bariana H.S., Kant L., Qureshi N., Forrest K., Miah H., Bansal U. (2022). Identification and characterisation of stripe rust resistance genes *Yr66* and *Yr67* in wheat cultivar VL Gehun 892. Agronomy.

[85] Zhang H.Q., Lang J., Ma S.Q., Zhang B.S. (2008). Genetic analysis and SSR mapping on a new stem stripe rust resistance gene *YrY206* in *Aegilops tauschii*. Chin. J. Biotechnol..

[86] Sun C., Zhang P., Fang Z.W., Zhang X., Yin J.L., Ma D.F., Zhu Y. (2019). Genetic analysis and molecular mapping of stripe rust resistance in an excellent wheat line Sanshumai1. J. Plant Pathol..

[87] Huang S., Liu S.J., Zhang Y.B., Xie Y.Z., Wang X.T., Jiao H.X., Wu S., Zeng Q., Wang Q., Singh R.P. (2021). Genome-wide wheat 55K SNP-based mapping of stripe rust resistance loci in wheat cultivar Shaannong 33 and their alleles frequencies in current Chinese wheat cultivars and breeding lines. Plant Dis..

[88] Rollar S., Geyer M., Hartl L., Mohler V., Ordon F., Serfling A. (2021). Quantitative trait loci mapping of adult plant and seedling resistance to stripe rust (*Puccinia striiformis* Westend.) in a multiparent advanced generation intercross wheat population. Front. Plant Sci..

[89] Marais G.F., Pretorius Z.A., Marais A.S., Wellings C.R. (2003). Transfer of rust resistance genes from *Triticum* species to common wheat. S. Afr. J. Plant Soil.

[90] Uauy C., Brevis J.C., Chen X.M., Khan I., Jackson L., Chicaiza O., Distelfeld A., Fahima T., Dubcovsky J. (2005). High-temperature adult-plant (HTAP) stripe rust resistance gene *Yr36* from *Triticum turgidum* ssp. dicoccoides is closely linked to the grain protein content locus Gpc-B1. Theor. Appl. Genet..

[91] Dong Z.Z., Hegarty J.M., Zhang J.L., Zhang W.J., Chao S.M., Chen X.M., Zhou Y., Dubcovsky J. (2017). Validation and characterization of a QTL for adult plant resistance to stripe rust on wheat chromosome arm 6BS (*Yr78*). Theor. Appl. Genet..

[92] Santra D.K., Chen X.M., Santra M., Campbell K.G., Kidwell K.K. (2008). Identification and mapping QTL for high-temperature adult-plant resistance to stripe rust in winter wheat (*Triticum aestivium* L.) cultivar Stephens. Theor. Appl. Genet..

[93] Bariana H.S., Bansal U.K., Schmidt A., Lehmensiek A., Kaur K., Miah H., Howes N., Mcintyre C.L. (2010). Molecular mapping of adult plant stripe rust resistance in wheat and identification of pyramided QTL genotypes. Euphytica.

[94] Liu L., Wang M.N., Feng J.Y., See D.R., Chao S.M., Chen X.M. (2018). Combination of all-stage and high-temperature adult-plant resistance QTL confers high-level, durable resistance to stripe rust in winter wheat cultivar Madsen. Theor. Appl. Genet..

[95] Wang Y., Hu Y., Gong F., Jin Y., Xia Y., He Y., Jiang Y., Zhou Q., He J., Feng L. (2022). Identification and mapping of QTL for stripe rust resistance in the Chinese wheat cultivar Shumai126. Plant Dis..

[96] Niu Z., Klindworth D.L., Friesen T.L., Chao S., Jin Y., Cai X., Xu S.S. (2011). Targeted introgression of a wheat stem rust resistance gene by DNA marker-assisted chromosome engineering. Genetics.

[97] Wu S., Pumphrey M.O., Bai G. (2009). Molecular mapping of stem rust resistance gene Sr40 in wheat. Crop Sci..

[98] Kosgey Z.C., Edae E.A., Dill-Macky R., Jin Y., Bulbula W.D., Gemechu A., Macharia G., Bhavani S., Randhawa M.S., Rouse M.N. (2021). Mapping and validation of stem rust resistance loci in spring wheat line CI 14275. Front. Plant Sci..

[99] Bajgain P., Rouse M.N., Bulli P., Bhavani S., Gordon T., Wanyera R., Njau P.N., Legesse W., Anderson J.A., Pumphrey M.O. (2015). Association mapping of North American spring wheat breeding germplasm reveals loci conferring resistance to Ug99 and other African stem rust races. BMC Plant Biol..

[100] Rouse M.N., Talbert L.E., Singh D., Sherman J.D. (2014). Complementary epistasis involving *Sr12* explains adult plant resistance to stem rust in Thatcher wheat (*Triticum aestivum* L.). Theor. Appl. Genet..

[101] Prins R., Dreisigacker S., Pretorius Z., van Schalkwyk H., Wessels E., Smit C., Bender C., Singh D., Boyd L.A. (2016). Stem rust resistance in a geographically diverse collection of spring wheat lines collected from across Africa. Front. Plant Sci..

[102] Sharma J.S., Overlander M., Faris J.D., Klindworth D.L., Rouse M.N., Kang H., Long Y., Jin Y., Lagudah E.S., Xu S.S. (2021). Characterization of synthetic wheat line Largo for resistance to stem rust. G3 Genes Genomes Genet..

[103] Jin Y., Szabo L.J., Rouse M.N., Fetch T., Pretorius Z.A., Wanyera R., Njau P. (2009). Detection of virulence to resistance gene *Sr36* within the TTKS race lineage of *Puccinia graminis* f. sp. *tritici*. Plant Dis..

[104] Bhavani S., Singh R.P., Argillier O., Huerta-Espino J., Singh S., Njau P., Brun S., Lacam S., Desmouceaux N., McIntosh R.A. (2011). Mapping durable adult plant stem rust resistance to the race Ug99 group in six CIMMYT wheats. Proceedings of the Borlaug Global Rust Initiative 2011 Technical Workshop.

[105] Njau P.N., Bhavani S., Huerta-Espino J., Keller B., Singh R.P. (2013). Identification of QTL associated with durable adult plant resistance to stem rust race Ug99 in wheat cultivar ‘Pavon 76’. Euphytica.

[106] Quraishi U.M., Pont C., Ain Q.U., Flores R., Burlot L., Alaux M., Quesneville H., Salse J. (2017). Combined Genomic and Genetic Data Integration of Major Agronomical Traits in Bread Wheat (*Triticum aestivum* L.). Front. Plant Sci..

[107] Letta T., Maccaferri M., Badebo A., Ammar K., Ricci A., Crossa J., Tuberosa R. (2013). Searching for novel sources of field resistance to Ug99 and Ethiopian stem rust races in durum wheat via association mapping. Theor. Appl. Genet..

[108] Genievskaya Y., Abugalieva S., Rsaliyev A., Yskakova G., Turuspekov Y. (2020). QTL mapping for seedling and adult plant resistance to leaf and stem rusts in Pamyati Azieva × Paragon mapping population of bread wheat. Agronomy.

[109] Maccaferri M., Harris N.S., Twardziok S.O., Pasam R.K., Gundlach H., Spannagl M., Ormanbekova D., Lux T., Prade V.M., Milner S.G. (2019). Durum wheat genome highlights past domestication signatures and future improvement targets. Nat. Genet..

[110] Singh R.P., McIntosh R.A. (1986). Cytogenetical studies in wheat. XIV. Sr8b for resistance to *Puccinia graminis* f. sp. *tritici*. Can. J. Genet. Cytol..

[111] Hailu E., Woldaeb G., Denbel W., Alemu W., Abebe T. (2015). Distribution of stem rust (*Puccinia graminis* f. sp. *tritici*) races in Ethiopia. Adv. Crop Sci. Tech..

[112] Randhawa M.S., Singh R.P., Dreisigacker S., Bhavani S., Huerta-Espino J., Rouse M.N., Nirmala J., Sandoval-Sanchez M. (2018). Identification and validation of a common stem rust resistance locus in two bi-parental populations. Front. Plant Sci..

[113] Peterson R.F., Campbell A.B., Hannah A.E. (1948). A diagrammatic scale for estimating rust intensity on leaves and stems of cereals. Can. J. Res..

[114] Roelfs A.P., Singh R.P., Saari E.E. (1992). Rust Diseases of Wheat: Concepts and Methods of Disease Management.

[115] Bansal U.K., Kazi A.G., Singh B., Hare R.A., Bariana H.S. (2014). Mapping of durable stripe rust resistance in a durum wheat cultivar Wollaroi. Mol. Breed..

[116] Taylor J., Butler D. (2017). R package AS Map, efficient genetic linkage map construction and diagnosis. J. Statist. Soft..

[117] Hussain W., Baenziger P.S., Belamkar V., Guttieri M.J., Venegas J.P., Easterly A., Sallam A., Poland J. (2017). Genotyping-by-Sequencing derived high-density linkage map and its application to QTL mapping of flag leaf traits in bread wheat. Sci. Rep..

[118] Manly K.F., Cudmore R.H., Meer J.M. (2001). Map Manager QTX.; cross-platform software for genetic mapping. Mamm. Genome.

[119] Wang S., Basten C.J., Zeng Z.B. (2011). Windows QTL Cartographer 2.5. Department of Statistics.

[120] Voorrips R. (2002). MapChart: Software for the graphical presentation of linkage maps and QTLs. J. Hered..

[121] Toth J., Pandurangan S., Burt A.J., Fetch J.M., Kumar S. (2019). Marker-assisted breeding of hexaploidy spring wheat in the Canadian Prairies. Can. J. Plant Sci..

[122] Wang Y., Zhang H., Xie J., Guo B., Chen Y., Zhang H., Lu P., Wu Q., Li M., Zhang D. (2018). Mapping stripe rust resistance genes by BSR-Seq: YrMM58 and YrHY1 on chromosome 2AS in Chinese wheat lines Mengmai 58 and Huaiyang 1 are *Yr17*. Crop J..

[123] He X., Kabir M.R., Roy K.K., Marza F., Chawade A., Duveiller E., Saint-Pierre C., Singh P.K. (2022). Genetic dissection for head blast resistance in wheat using two mapping populations. Heredity.

