# Supplementary material for: Genetic Dissection of Triple Rust Resistance (Leaf, Yellow, and Stem Rust) in Kenyan Wheat Cultivar, “Kasuku”
Source: Plants (Basel). 2025 Mar 23;14(7):1007. doi: 10.3390/plants14071007 (PMC11990868; doi:10.3390/plants14071007)
Supplement: Supplementary file 1 [file plants-14-01007-s001.zip › plants-3467131-supplementary.pdf]

Supplementary material

Genetic dissection of triple rust resistance in Kenyan wheat cultivar, "Kasuku"

Naeela Qureshi [1]*, Ravi Prakash Singh [1] and Sridhar Bhavani [2]

[1] International Maize and Wheat Improvement Center (CIMMYT), Carretera Mexico-Veracruz Km. 45, El-Batan, Texcoco 56237, Mexico
[2] International Maize and Wheat Improvement Center (CIMMYT), ICRAF Campus, United Nations Avenue, Gigiri, Nairobi P.O. Box 1041-00621, Kenya

Table S1. Summary of Identified Genetic Loci for Stripe Rust, Leaf Rust, and Stem Rust in the Kasuku/Apav#1 Recombinant Inbred Line (RIL) Population, including LOD Scores, Phenotypic Variance Explained (R2), and Physical Positions (Mb).

QTL	Year	Environment	Marker interval	Position	LOD score	*R2 (%)*
QLrYrSr.cim-1BL	LR19-1	Obregon, Mexico	100107511-2260122	678-685 Mb	11.5	12
	LR19-2		1160899-2260122	678-685 Mb	22.7	19
	LR20	El-Batan, Mexico	1128427-1049014	678-685 Mb	23.59	18
	LR21-1	El-Batan, Mexico	1160899-2260122	678-685 Mb	18.7	16
	LR21-2		1160899-2260122	678-685 Mb	12	9
	LR22-1	Obregon, Mexico	100107511-1160899	678-685 Mb	26.9	33
	LR22-2		100107511-1160899	678-685 Mb	9.4	8
	YR20-1	Toluca, Mexico	100107511-1160899	678-685 Mb	14.3	8
	YR20-2		100107511-1160899	678-685 Mb	13	10
	YR21-1	Toluca, Mexico	100107511-1160899	678-685 Mb	7.5	6
	YR21-2		100107511-1160899	678-685 Mb	10.4	9
	YRKEN21-1	Njoro, Kenya	100107511-1160899	678-685 Mb	7	13
	YRKEN21-2		10592752-1049014	678-685 Mb	11	20
	YRKEN21-3		10592752-1049014	678-685 Mb	16.4	23
	SR-21-1	Njoro, Kenya	100107511-1160899	678-685 Mb	4.64	8
	SR-21-2		100107511-1160899	678-685 Mb	4.4	4

	SR-21-3		100107511-1160899	678-685 Mb	10	15
	SR-23-1	Njoro, Kenya	1109580-3534344	609-614 Mb	4	5
	SR-23-2		1109580-3534344	609-614 Mb	5	5.5
QLrYr.cim-2AS	LR19-1	Obregon, Mexico	1109148a1-1109165a1	~ 8 Mb	21.7	27
	LR19-2		1109148a1-1109165a1	~ 8 Mb	20	27
	LR21-1	El-Batan, Mexico	7347451-1109165a1	~ 8 Mb	30	32
	LR21-2		7347451-1109165a1	~ 8 Mb	34.7	38
	LR22-1	Obregon, Mexico	1109148a1-1109165a1	~ 8 Mb	14	15
	LR22-2		1109148a1-1109165a1	~ 8 Mb	21.5	21
	YR20-1	Toluca, Mexico	1109148a1-1109165a1	~ 8 Mb	53	56
	YR20-2		1109148a1-1109165a1	~ 8 Mb	47.7	59
	YR21-1	Toluca, Mexico	7347451-1109165a1	~ 8 Mb	41.7	54
	YR21-2		7347451-1109165a1	~ 8 Mb	40.5	45
	YR191	Toluca, Mexico	7347451-1109165a1	~ 8 Mb	41.7	54
QLrYr.cim-3AL	LR19-1	Obregon, Mexico	7334423-10098678	~ 747 Mb	3	5.3
	LR19-2		7334423-10098678	~ 747 Mb	3.1	7.09
	LR20	El-Batan, Mexico	7334423-10098678	~ 747 Mb	3	6.8
	LR21-1	El-Batan, Mexico	7334423-10098678	~ 747 Mb	3	5.09
	LR21-2		7334423-10098678	~ 747 Mb	3	7
	LR22-1	Obregon, Mexico	7334423-10098678	~ 747 Mb	3	6.2
	LR22-2		7334423-10098678	~ 747 Mb	3	6.6
	YR20-1	Toluca, Mexico	7334423-10098678	~ 747 Mb	4.6	8.8
	YR20-2		7334423-10098678	~ 747 Mb	3.5	8.6
	YR21-1	Toluca, Mexico	7334423-10098678	~ 747 Mb	4	7.2
	YR21-2		7334423-10098678	~ 747 Mb	3.7	8.5
QLrYrSr.cim-6AL	LR19-1	Obregon, Mexico	1109580-3534344	609-614 Mb	6.3	7.3
	LR19-2		1109580-3534344	609-614 Mb	7	10.2
	LR20	El-Batan, Mexico	1109580-3534344	609-614 Mb	5.8	9.98
	LR21-1	El-Batan, Mexico	1109580-3534344	609-614 Mb	6.42	7.7
	LR21-2		1109580-3534344	609-614 Mb	6.7	10.95
	LR22-1	Obregon, Mexico	1109580-3534344	609-614 Mb	7.2	9.9

	LR22-2		1109580-3534344	609-614 Mb	7	11.74
	YR20-1	Toluca, Mexico	1109580-3534344	609-614 Mb	5.5	9.5
	YR20-2		1109580-3534344	609-614 Mb	4	8.62
	YR21-1	Toluca, Mexico	1109580-3534344	609-614 Mb	4.74	7.52
	YR21-2		1109580-3534344	609-614 Mb	5.58	9.59
	YRKEN21-1	Njoro, Kenya	1109580-3534344	609-614 Mb	4	4
	YRKEN21-2		1109580-3534344	609-614 Mb	3.7	6.36
	YRKEN21-3		1109580-3534344	609-614 Mb	3.4	3.9
	YRKEN23-1	Njoro, Kenya	1109580-3534344	609-614 Mb	4.5	4
	YRKEN23-2		1109580-3534344	609-614 Mb	4	5.5
	SR-21-1	Njoro, Kenya	1109580-3534344	609-614 Mb	4.39	6
	SR-21-2		1109580-3534344	609-614 Mb	5.6	5
	SR-21-3		1109580-3534344	609-614 Mb	6.75	7.68
	SR-23-1	Njoro, Kenya	1109580-3534344	609-614 Mb	4	5
	SR-23-2		1109580-3534344	609-614 Mb	5	5.5
QLr.cim-2DS	LR19-1	Obregon, Mexico	1217169-991048	12-17 Mb	7.5	9
	LR19-2		1010209-3024321	12-17 Mb	8.5	8
	LR20	El-Batan, Mexico	1217169-100065351	12-17 Mb	7.7	5
	LR21-1	El-Batan, Mexico	1092168-991048	12-17 Mb	7.7	6
	LR21-2		1092168-991048	12-17 Mb	8.5	7
	LR22-2	Obregon, Mexico	1092168-991048	12-17 Mb	3.6	4
QLr.cim-6DS	LR19-1	Obregon, Mexico	100095461-3034448	7-19 Mb	3.6	5.5
	LR19-2		100095461-3034448	7-19 Mb	3.6	7.5
	LR20	El-Batan, Mexico	100095461-3034448	7-19 Mb	3	7.1
	LR21-1	El-Batan, Mexico	100095461-3034448	7-19 Mb	3	5
	LR21-2		100095461-3034448	7-19 Mb	3.11	7.59
	LR22-1	Obregon, Mexico	1116149-3034448	7-19 Mb	4.15	7.88
	LR22-2		1116149-3034448	7-19 Mb	4.2	9.01
QYrKen.cim-3DS	YRKEN21-2	Njoro, Kenya	1071002-4993778	~ 150 Mb	3.4	6.3

	YRKEN21-3		1071002-4993778	~ 150 Mb	4.7	7.8
	YRKEN23-1	Njoro, Kenya	1071002-4993778	~ 150 Mb	4.5	7
QYrKen.cim-6BS	YRKEN23-2		1071002-4993778	~ 150 Mb	5	7.5
	YRKEN21-2	Njoro, Kenya	1116951-3941697	87-120 Mb	4	7
	YRKEN21-3		1116951-3941697	87-120 Mb	7.2	10
	YRKEN23-1	Njoro, Kenya	1116951-3941697	87-120 Mb	5	7.5
	YRKEN23-2		1116951-3941697	87-120 Mb	6.5	9
QSr.cim-2BS	SR21-1	Njoro, Kenya	1026962-3021198	68-76 Mb	3.2	7.1
	SR21-2		1026962-3021198	68-76 Mb	4.8	9.3
	SR21-3		1026962-3021198	68-76 Mb	4.7	7.5
	SR23-1	Njoro, Kenya	1026962-3021198	68-76 Mb	4	7
	SR23-2		1026962-3021198	68-76 Mb	4.2	8.5
QSr.cim-5AL	SR21-2	Njoro, Kenya	1200952-100101071	682 Mb	4	7
	SR21-3		1200952-100101071	682 Mb	5	7
	SR23-1	Njoro, Kenya	1200952-100101071	682 Mb	4.1	7.1
	SR23-2		1200952-100101071	682 Mb	4	7
QSr.cim-6AS	SR21-1	Njoro, Kenya	3025616-3222462	9-10 Mb	4.7	9
	SR21-2		3025616-3222462	9-10 Mb	5.9	12
	SR23-1	Njoro, Kenya	3025616-3222462	9-10 Mb	4.7	9
	SR23-2		3025616-3222462	9-10 Mb	5.9	12
